# The neuropeptidergic connectome of *C. elegans*

**DOI:** 10.1016/j.neuron.2023.09.043

**Published:** 2023-11-15

**Authors:** Lidia Ripoll-Sánchez, Jan Watteyne, HaoSheng Sun, Robert Fernandez, Seth R. Taylor, Alexis Weinreb, Barry L. Bentley, Marc Hammarlund, David M. Miller, Oliver Hobert, Isabel Beets, Petra E. Vértes, William R. Schafer

**Affiliations:** 1 Neurobiology Division, MRC Laboratory of Molecular Biology, Cambridge, UK; 2 Department of Psychiatry, Cambridge University, Cambridge, UK; 3 Department of Biology, KU Leuven, Leuven, Belgium; 4 Department of Biological Sciences/HHMI, Columbia University, New York, NY, USA; 5 Department of Cell, Developmental, and Integrative Biology, University of Alabama at Birmingham, Birmingham, AL, USA; 6 Department of Cell and Developmental Biology, Vanderbilt University School of Medicine, Nashville, TN, USA; 7 Departments of Genetics and Neuroscience, Yale University School of Medicine, New Haven, CT, USA; 8 Cardiff School of Technologies, Cardiff Metropolitan University, Cardiff, UK

**Keywords:** neuropeptides, connectomics, networks, neuromodulation, C. elegans

## Abstract

Efforts are ongoing to map synaptic wiring diagrams, or connectomes, to understand the neural basis of brain function. However, chemical synapses represent only one type of functionally important neuronal connection; in particular, extrasynaptic, “wireless” signaling by neuropeptides is widespread and plays essential roles in all nervous systems. By integrating single-cell anatomical and gene-expression datasets with biochemical analysis of receptor-ligand interactions, we have generated a draft connectome of neuropeptide signaling in the *C. elegans* nervous system. This network is characterized by high connection density, extended signaling cascades, autocrine foci, and a decentralized topology, with a large, highly interconnected core containing three constituent communities sharing similar patterns of input connectivity. Intriguingly, several key network hubs are little-studied neurons that appear specialized for peptidergic neuromodulation. We anticipate that the *C. elegans* neuropeptidergic connectome will serve as a prototype to understand how networks of neuromodulatory signaling are organized.

## Highlights

• A comprehensive map of neuropeptide signaling in *C. elegans* has been assembled
• Neuropeptide signaling forms a dense and decentralized wireless network
• The peptidergic network core exhibits mesoscale structure and distinct hubs
• Neuropeptides link disconnected neural circuits to the connectome

## Graphical abstract

**Figure:**
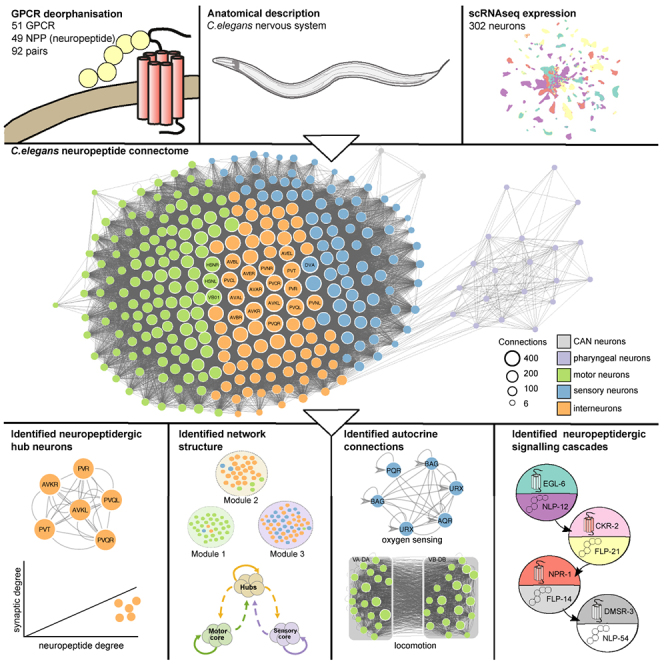

## Teaser

Neuromodulation by peptides is essential for brain function. By comprehensively mapping neuropeptide signaling in the nematode *C. elegans*, Ripoll-Sánchez et al. define a dense wireless network whose organization differs in important ways from wired brain circuits. This network is a prototype for understanding neuropeptide signaling networks in larger brains.

## Introduction

Understanding how behavior arises from neuronal interactions in the brain is one of the great challenges of modern neuroscience. Recently, efforts have begun to map the synaptic wiring diagrams, or connectomes, of diverse nervous systems, including *Drosophila*, *Platynereis*, *Ciona*, zebrafish, and mouse.^1^^,^^2^^,^^3^^,^^4^^,^^5^^,^^6^^,^^7^ This has involved reconstructing volumes of brain tissue using electron microscopy (EM) image volumes to trace neuronal processes and identify hallmarks of chemical synapses between identified neurons.

Although most connectomics research has focused on synaptic connectivity, chemical synapses are not the only means by which neurons communicate. For example, many important interactions between neurons involve volume transmission, where extrasynaptically secreted molecules activate receptors on neurons often synaptically unconnected to the releasing neuron. Unlike synaptic transmission, where signaling is restricted to neurons on either side of the synapse, volume transmission can mediate signaling across distances of microns.^8^^,^^9^^,^^10^ Such extrasynaptic signaling has been described for classical neurotransmitters as well as monoamines and is particularly prevalent for neuropeptides, which are released from dense-core vesicles and act on longer temporal and spatial scales.^10^^,^^11^ These “wireless” interactions play key roles in neural circuits and are thus critical for understanding the neuronal basis of behavior.^12^^,^^13^^,^^14^^,^^15^^,^^16^^,^^17^^,^^18^

Neuropeptides are ancient and conserved signaling molecules that mediate important functions in the brains of all organisms.^19^^,^^20^^,^^21^^,^^22^^,^^23^ Biologically active peptides are usually enzymatically processed from larger polypeptide precursors^24^ and typically bind to G protein-coupled receptors (GPCRs) that modulate diverse aspects of neuronal physiology.^25^ Neuropeptide-activated GPCRs subdivide into rhodopsin-like (class A) and secretin-like (class B) families; within these groups, many receptors (e.g., oxytocin/vasopressin, neuropeptide Y/F, neuromedin U, and somatostatin) are conserved across animal phyla.^26^^,^^27^ Neuropeptide systems play conserved roles in the control of behavioral states, including those involved in feeding, sleep, arousal, reproduction, and learning.^14^^,^^28^^,^^29^^,^^30^^,^^31^^,^^32^^,^^33^ In humans, neuropeptide receptors have become sought-after targets for new neuropsychiatric treatments; 50 drugs targeting peptidergic GPCRs have been approved by the FDA,^34^ including orexin antagonists for treating insomnia,^35^ a substance P antagonist for chemotherapy-induced nausea,^36^ and a GIP agonist for Alzheimer’s and Parkinson’s diseases.^37^ The many additional human peptide GPCRs^18^^,^^38^ hold further untapped therapeutic potential.

The diversity and extent of neuropeptide signaling implies that the pathways for peptidergic communication can also be considered as a network. The genomes of all animals encode many, often hundreds, of neuropeptides, along with a similarly large number of GPCRs.^18^^,^^39^^,^^40^^,^^41^ Moreover, in contrast to monoamines, typically expressed in small subsets of neurons, neuropeptides are expressed broadly; recent transcriptomic studies indicate that most if not all neurons in the mouse cerebral cortex express several neuropeptides as well as multiple neuropeptide-binding GPCRs.^39^^,^^42^ This implies that peptidergic signaling underpins a dense and pervasive interaction network involving most of the nervous system.^43^^,^^44^ Because neuropeptide signaling is thought to be mostly extrasynaptic, the topology and structure of these wireless peptidergic connectomes may be fundamentally distinct from those of wired synaptic connectomes.^45^ However, in most organisms there is insufficient data to map the structure of these networks; even where detailed transcriptomic data exists, the anatomical positions and synaptic connectivities of neuropeptide and receptor-expressing neurons are imprecisely known, precluding the juxtaposition of peptidergic signaling maps with wired synaptic connectomes.

The nematode *C. elegans* is an attractive animal with which to investigate the organization of neuropeptide signaling networks. *C. elegans* was the first organism with a completely mapped synaptic neuronal connectome, with each of its 302 neurons and approximately 2,300 synaptic connections between them identified through EM reconstructions.^46^^,^^47^^,^^48^^,^^49^ Despite its small size, the *C. elegans* nervous system shares structural features in common with those of larger animals. For example, the *C. elegans* connectome, like those of larger nervous systems, exhibits a small-world topology, with relatively high clustering paired with relatively short average path lengths^50^^,^^51^ Likewise, the nematode nervous system is modular, with functionally segregated local clusters of high within-group connectivity.^52^^,^^53^^,^^54^^,^^55^ Finally, the worm connectome contains a small number of highly connected hubs interconnected in a core or rich club that facilitates communication between modules.^56^ Similar rich-club topology has been observed in bigger brains, including the human cortex.^57^^,^^58^ Shared features are also apparent at the microcircuit level; for example, feed-forward motifs are overrepresented in both the nematode connectome and the mammalian cortex.^59^^,^^60^^,^^61^ Thus, insights gained from analysis of neuropeptide signaling networks in *C. elegans* may reveal organizational principles conserved in larger brains.

Although the *C. elegans* nervous system is anatomically small, its neuropeptide signaling pathways show remarkable biochemical complexity. Its genome contains at least 159 predicted neuropeptide precursor (NPP) genes producing over 300 different peptides^62^^,^^63^ Approximately 150 genes encode known or predicted peptide-activated GPCRs,^64^ numbers similar to the human genome.^18^^,^^65^ Each *C. elegans* neuron expresses a unique combination of neuropeptide-encoding genes,^40^ like vertebrate neurons.^18^^,^^39^^,^^40^^,^^42^^,^^43^ Many *C. elegans* neuropeptides and cognate receptors are phylogenetically conserved across phyla and have clear human homologs, with some families (such as RFamide peptides) having undergone expansion in the nematode lineage.^66^ Thus, neuropeptide signaling in nematodes shows surprising conservation and similar diversity to neuropeptide signaling in the human brain, despite vast differences in neuron number and anatomical complexity.

Here, we present a draft connectome of neuropeptidergic signaling in *C. elegans*, built by integrating gene expression, ligand-receptor interaction, and anatomical datasets.^40^^,^^66^ The novel structure and topology of this neuropeptide connectome serves as a prototype for understanding the brain-wide organization of peptidergic signaling in a whole organism.

## Results

### Mapping the neuropeptidergic connectome

**Figure 1:**
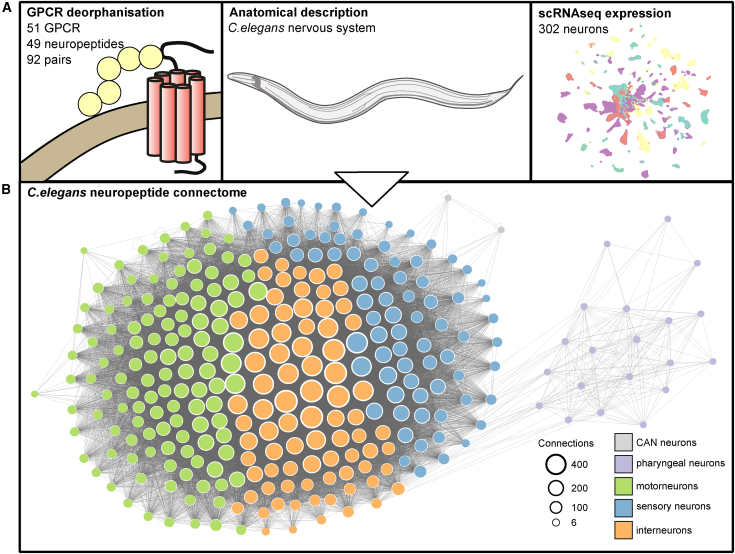
Construction of the neuropeptidergic connectome (A) Datasets used to build the network, including neuropeptide-GPCR interaction data,^66^ nervous system anatomy,^49^ and single-neuron expression data.^40^ (B) Graphical representation of the resulting neuropeptide connectome; all neurons form a single connected network.

To generate a neuropeptide connectome, we integrated information from biochemical, gene expression, and anatomical datasets^40^^,^^66^ to infer potential pathways for neuropeptide signaling between individual *C. elegans* neurons (Figure 1A). In constructing the network, we considered two neurons (nodes) to be connected if the first neuron expresses a particular neuropeptide ligand and the second expresses a paired receptor, subject to spatial constraints on signaling (Figure 1B). Thus, for each dataset, it was necessary to threshold for biologically relevant interactions.

To identify biologically relevant molecular interactions between neuropeptides and receptors, we used data from a large-scale reverse pharmacology screen that tested over 55,384 potential neuropeptide-receptor interactions *in vitro*,^66^ identifying 461 neuropeptide-receptor pairs showing concentration-dependent activation with a half-maximal effective concentration (EC_50_) between 0.1 pM and 22 μM.^66^ Genetically, these pairs are encoded by 55 neuropeptide and 56 GPCR genes, with 148 unique gene-gene interactions. The ligand-receptor interactions were complex: several peptides activated more than one receptor (versatile peptides) and several receptors were activated by peptides encoded by multiple precursor genes (promiscuous receptors).^66^ In assessing these neuropeptide-receptor pairings for biological relevance, we initially opted for an EC_50_ threshold of 500 nM, as neuropeptide-receptor couples with EC_50_ values in this range have been validated *in vivo*.^14^^,^^32^^,^^66^^,^^67^^,^^68^ By this criterion, we defined 92 individual neuropeptide-receptor gene couples, with a large number (51) of the predicted neuropeptide GPCRs having at least one identified ligand. For some analyses, we also considered a stricter EC_50_ threshold (100 nM) to evaluate the sensitivity of our results to this parameter (Figures S7 and S12).

**Figure 2:**
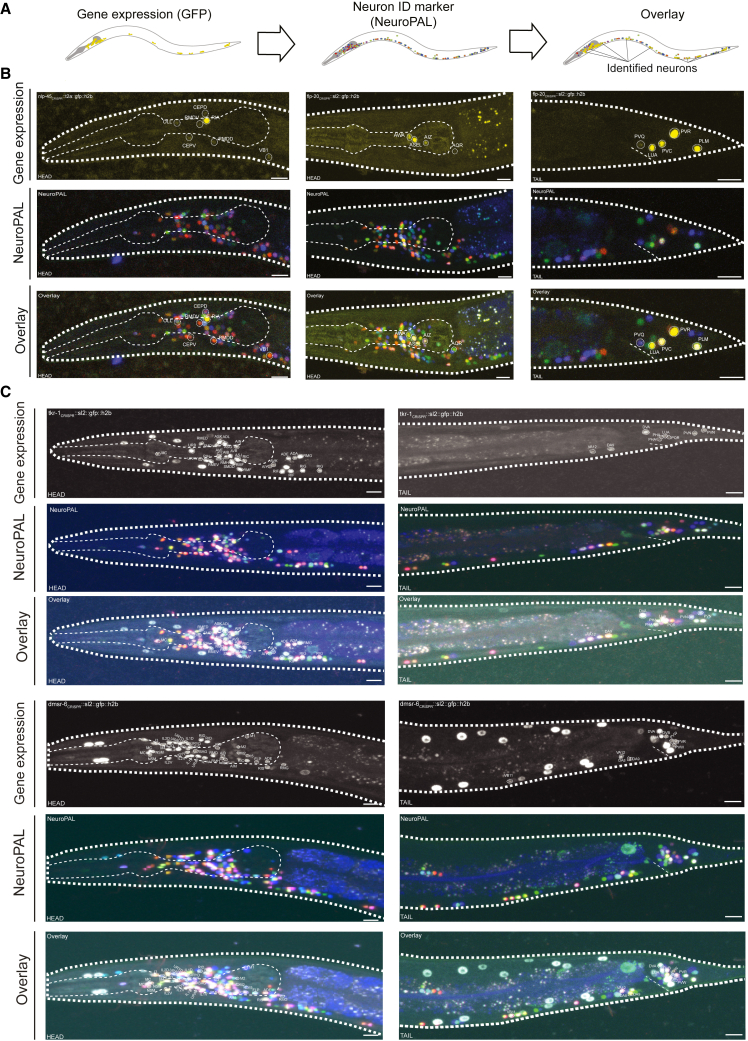
Assessment of gene expression thresholds using single-copy knockin reporters GFP-positive neurons were identified using the NeuroPAL multicolor transgene.^69^ Segments showing neuronal expression are pictured; individual neurons are labeled. Scale bars represent 10 μm. (A) Strategy for neuron identification. Reporter expression is overlaid with the multicolor NeuroPAL expression pattern, allowing neuron identification. (B) Reporters for representative NPP genes *nlp-45* (left) and *flp-20* (right). (C) Reporters for representative GPCR genes *tkr-1* (above) and *dmsr-6* (below). Images for the complete set of 17 NPP and nine GPCR reporters are in Figure S1.

To determine which neurons express each neuropeptide and receptor, we used single-neuron transcriptome data from the CeNGEN (*C. elegans* Neuronal Gene Expression Map & Network) project, describing single-cell RNA sequencing (scRNA-seq) profiles of all predicted neuropeptide and peptide-activated GPCR genes in *C. elegans*.^40^ These data were differentially thresholded across the nervous system based on a ground-truth dataset of reliable gene expression using fosmid or receptor-tagged reporters.^40^ To determine the most appropriate threshold for neuropeptide and receptor expression, we obtained single-copy genomic knockin reporters for 17 representative NPPs and 9 representative peptidergic GPCRs and characterized their expression patterns comprehensively using the NeuroPAL (Neuronal Polychrome Atlas of Landmarks) marker strain^69^ (Figures 2A and 2B). For both peptides and receptors, we found that the most stringent threshold was a good approximation of the expression pattern seen in reporter lines (Figure 2; Tables S1, S2, and S3). Although this strict threshold has lower discovery power and thus may undercount how many neurons express each gene, its stringency minimizes the likelihood that our network would contain edges that do not represent authentic paths for neuropeptide signaling.

### Evaluating spatial constraints on neuropeptide signaling

**Figure 3:**
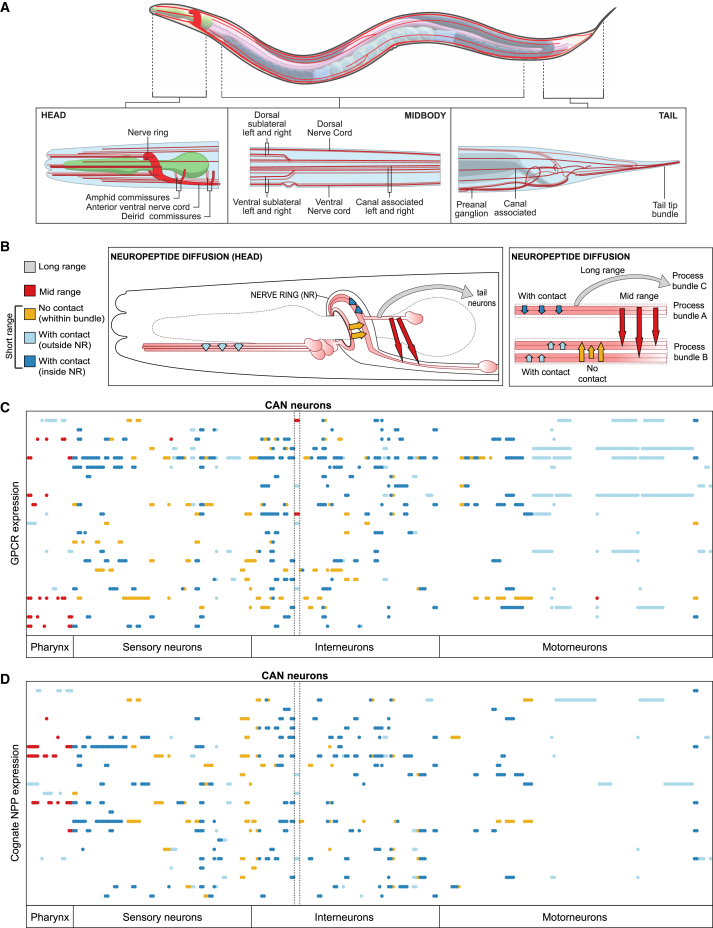
Assessment of the spatial scale of neuropeptide signaling (A) Anatomical overview of the *C. elegans* hermaphrodite nervous system. Neuronal bundles are represented in red and the pharynx in green. (B) Details of assessed diffusion models. Contact interactions are defined as occurring between neurons with processes in the same nerve ring stratum^72^^,^^73^ or small process bundle. Short-range connections include interactions within the same neuronal bundle. Mid-range connections occur between different bundles within the same body region, i.e., head, midbody, or tail. Long-range connections are between neurons in different body regions. (C) Expression matrix for 23 GPCRs, activated by a single ligand that activates no other receptor.^66^ Columns indicate neurons, sorted by type; each row indicates a GPCR. Colors indicate the diffusion range required for communication with at least one ligand-expressing neuron: blue indicates contact interactions within a nerve ring stratum (dark blue) or a thin neuronal bundle (light blue); mustard indicates short-range connections between nerve-ring strata; red indicates mid-range connections between neurons in different bundles. (D) Expression matrix for the 23 cognate neuropeptide precursor genes for the receptors in (C). Neurons are sorted by type on the x axis; neuropeptide genes are on the y axis. Colors indicate the diffusion range required for communication with a receptor-expressing neuron, as described above. GPCR and NPP identities are included in Figure S2.

These biochemical and gene expression datasets allowed us to infer which neurons express neuropeptide and receptor genes that could mediate a neuromodulatory interaction; however, neuroanatomy and the physics of diffusion might constrain some of these interactions *in vivo*. We used EM anatomical reconstructions,^49^^,^^70^^,^^71^ defining for each neuron its anatomical location and the neuropil bundles containing its axons and dendrites^49^^,^^71^ to assess four possible models for diffusion of neuropeptides to their target receptors (Figures 3A and 3B; Table S4). In the first, most permissive model, long-range signaling is permitted, and neuropeptidergic connections can take place between any neuron pair. In the second (“mid-range”) model, neuropeptidergic connections can occur only between neuronal processes in the same anatomical area, such as the head, tail, and midbody (Figures 3A and 3B). The third (“short-range”) model only allowed peptidergic connections between neurons whose processes overlap in the same process bundle (e.g., nerve ring, ventral cord). Finally, because the nerve ring, into which most neurons project, can be divided into four layers or strata based on patterns of physical contact,^72^^,^^73^ we also considered a fourth model in which neuropeptide signaling inside the nerve ring is constrained within individual strata and thus between neurons in physical contact.

To evaluate each model, we investigated whether the expression of receptors and ligands was consistent with its restrictions on neuropeptide diffusion (Figures 3C and 3D). To simplify this analysis, we focused on neuropeptide-receptor couples where the receptor has only one ligand and the neuropeptide only binds to that receptor, assessing whether, under each model’s constraints, all receptor-expressing neurons can receive a signal from neurons expressing its ligand. For example, if neuropeptide signaling occurred only between neurons in physical contact (model 4) we would expect neurons expressing a given receptor to always make contact with neurons expressing its ligand. We likewise analyzed ligand expression in the same manner, asking whether under a given model there were ligand-expressing neurons that could not communicate with cells expressing its receptor.

This analysis argued against the most restrictive model in which ligands cannot diffuse between nerve ring strata. We observed many examples where a neuropeptide receptor was expressed in strata that did not express its ligand (Figures 3B and 3C, orange points). For instance, while the *capa-1* NPP gene is expressed only in ASG neurons, whose axons are in stratum 4, its receptor NMUR-1 is expressed in all strata.^32^ Thus, NMUR-1 receptors in strata 1–3 must be activated by CAPA-1 peptides that diffuse from stratum 4. Overall, 21 of 23 receptors analyzed were expressed in at least one neuron making no contact with a neuron expressing its ligand. Likewise, 20 of the 23 NPP genes were expressed in neurons making no contact with neurons expressing its receptor (Figure 3D). Because the nerve ring strata are not separated by glial or other barriers, and calcium imaging studies^14^^,^^74^ indicate that neuropeptides can indeed travel between strata, it is likely that neuropeptides are not constrained within neuronal bundles.

We likewise observed cases in which NPP and receptor expression implies mid-range signaling between nerve bundles (Figures 3B and 3C). For example, the *frpr-7* receptor is expressed in multiple pharyngeal neurons, although its ligand FLP-1 is released exclusively from AVK neurons, whose processes lie in the nerve ring and ventral cord.^13^ Thus, FRPR-7 receptors in the pharynx appear to be activated by peptides from the nerve ring, consistent with published evidence that neuropeptides can signal between head and pharyngeal neurons.^13^^,^^75^ Indeed, for a majority (7/13) of receptors expressed in pharyngeal neurons, their ligand was expressed only outside the pharynx (Figure 3C). Likewise, receptors such as NPR-3 and TRHR-1 are expressed in the CAN neurons, yet their ligands FLP-15 and NLP-54 are only expressed outside the canal-associated nerve^76^ (Figures 3C and 3D). Thus, we hypothesize that some neuropeptide signaling occurs between different nerve bundles, in particular between the pharynx and the nerve ring and between CAN and other somatic nerves. We therefore focused our subsequent analysis on the mid-range model 2, the most conservative model consistent with all receptor and NPP gene expression, as well as the short-range model 3, which accounts for all gene expression outside the pharynx and CAN. Conversely, because model 4 is likely to exclude many biologically relevant short-range interactions, while model 1 includes many long-range interactions of uncertain biological relevance, we did not study these networks in detail.

### Neuropeptide networks exhibit diverse topologies

**Figure 4:**
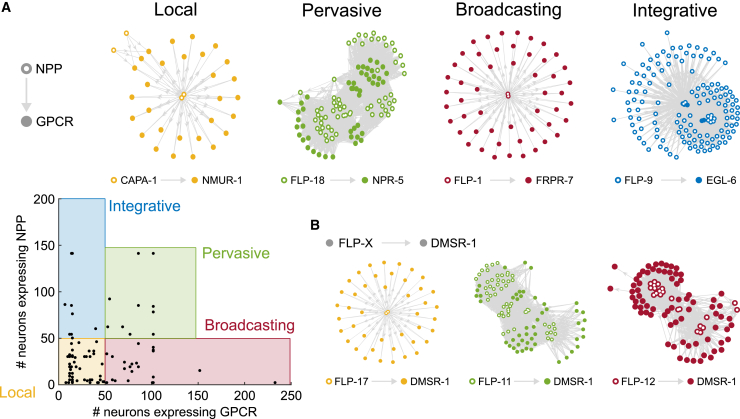
Individual NPP-GPCR networks exhibit different topologies (A) Classification of individual peptidergic networks based on NPP and receptor expression domains. Bottom left: scatterplot showing the number of neurons expressing a particular GPCR versus the neurons expressing the corresponding NPP gene for each of 92 individual networks. Local networks show restricted NPP and GPCR expression (≤50 neurons). Pervasive networks have broad NPP and GPCR expression (>50 neurons), broadcaster networks show broad GPCR (˃50 neurons) but restricted NPP expression (≤50 neurons), and integrative networks display broad NPP (˃50 neurons) and restricted GPCR expression (≤50 neurons). Filled circles indicate receptor expression; empty circles indicate neuropeptide expression. Example graphs: local network CAPA-1/NMUR-1,^32^ pervasive network FLP-18/NPR-5,^77^ broadcaster network FLP-1/FRPR-7,^13^ and integrative network NLP-47/GNRR-1.^78^ (B) Examples of networks using common receptors with different topologies, depending on the peptide ligand. Graphs of all NPP-GPCR pairs are in Figure S4.

Based on these criteria, we constructed network graphs between neuropeptide-expressing and receptor-expressing neurons for each of the 92 individual neuropeptide/receptor couples in our dataset (Figures S3–S6). These networks were filtered by removing edges between neurons without processes in the same bundle (for short-range networks, Figures S3 and S4) or the same body region (for mid-range networks, Figures S5 and S6). In their short-range versions, 78 of these ligand-receptor couples formed single connected networks, whereas 13 formed networks with two or three disconnected components (Figures 4 and S4). In their mid-range versions, all 92 couples formed single connected networks (Figure S6). Salient features of these networks (summarized in Table S5 and http://github.com/LidiaRipollSanchez/Neuropeptide-Connectome) can be further explored at http://www.nemamod.org/.

We observed a diverse range of topologies in these networks for individual peptide-receptor couples. In particular, networks varied in assortativity, or the extent to which nodes connect preferentially to nodes of similar degree. Using the short-range model, we observed that most of the couples formed local networks in which both ligand and receptor were expressed in restricted sets of neurons (Figures 4A and S4). These networks showed relatively low average degree and encompassed only a subset of the nervous system. In addition, we found eight highly disassortative *integrative* networks, with many low-degree peptide-releasing neurons signaling to relatively few high-degree receptor-expressing neurons (Figures 4A and S4). We also observed 23 disassortative *broadcasting* networks, characterized by a small number of high-degree peptide-releasing neurons signaling to many low-degree receiving neurons with broadly expressed receptors (Figures 4A and S4). Interestingly, both integrative and broadcasting networks involved promiscuous receptors, which also figured prominently in a fourth category of more assortative, *pervasive* networks, where both ligand and receptor show broad expression and most neurons exhibit high degree (Figures 4A and 4B). Relaxing spatial restrictions on peptide diffusion had modest impact on network topology, with four networks that were local in the short-range model becoming integrative (one network) or broadcasting (three networks) in the mid-range model (Figure S6). Thus, the topologies of neuropeptide networks appear relatively robust to assumptions about the spatial scope of neuropeptidergic signaling.

### The neuropeptide connectome is a decentralized, dense network

**Figure 5:**
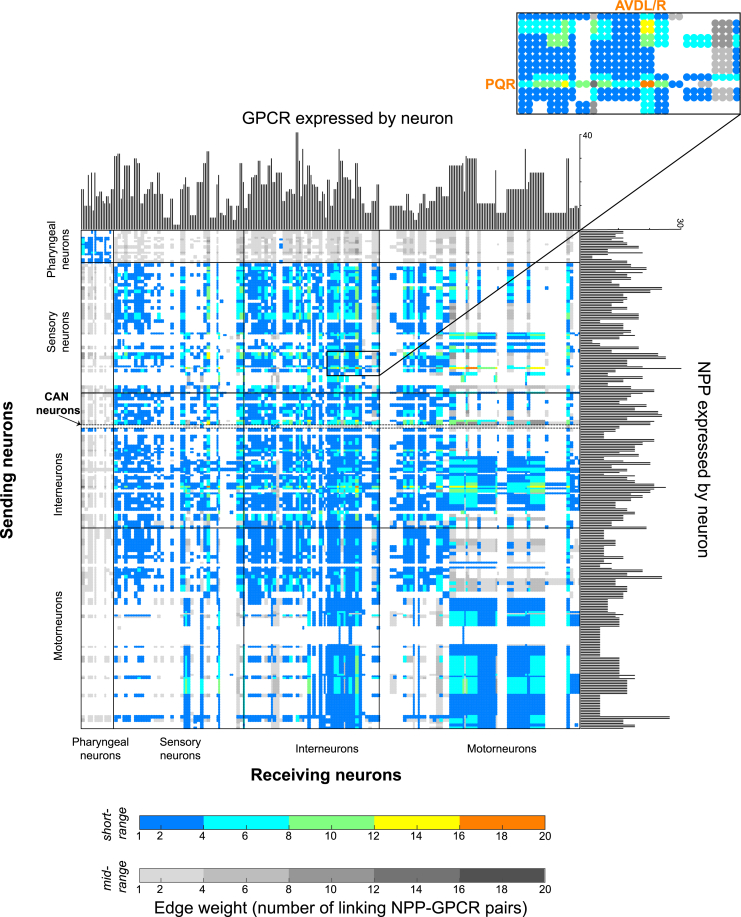
The aggregate neuropeptide connectome connects all neurons in a dense network Shown is the adjacency matrix of the aggregate network using short-range (color) and mid-range (gray) diffusion models. Histograms on the axes represent numbers of NPP and GPCR genes per neuron. Edge weights (range: 1–18) indicate the number of different NPP-GPCR pathways connecting a neuron pair in a given direction. 5% of all connections are putative autocrine connections.

By aggregating the networks from the individual neuropeptide-receptor couples, we next compiled complete neuropeptide connectomes based on short-range or mid-range signaling (Figure 5). Even the conservative short-range network exhibited high connection density, with more than a third of all possible connections or edges (0.3437) present in the network. Allowing mid-range connections (0.4429) increased this density further (Figure S9B). By comparison, the *C. elegans* synaptic (0.0251) and monoamine (0.0236) networks were far less dense. We also computed edge weights for the neuropeptide networks based on the number of neuropeptide-receptor pairs capable of signaling between nodes. Although a large number (35% in the short-range network) of neuron pairs were connected by a single neuropeptide-receptor interaction, some (9%) were connected by ≥6 different peptide-receptor couples (Figure 5). In the most extreme case, the AVD interneurons and the PQR oxygen-sensing neurons were linked by 18 different neuropeptide-receptor pairs, suggesting extraordinarily complex patterns of signaling between these cells (Figure 5). Other high-weight, biochemically complex connections also occurred in the oxygen-sensing circuit and were mediated by a common set of promiscuous receptors (DMSR-1, DMSR-7, and FRPR-8) and versatile neuropeptides (FLP-4, FLP-9, FLP-10, and FLP-13). Such connection of neurons by multiple neuropeptidergic channels may allow complex regulation by context and experience.

Collectively, the connections mediated by neuropeptide signaling showed only modest overlap with those mediated by synapses. For example, 1,522 neuron pairs are connected by both chemical synapses and peptidergic interactions; this represents only 5% and 4%, respectively, of total neurons with inferred short- or mid-range peptidergic interactions. A striking consequence of this extensive extrasynaptic peptidergic signaling, for the mid-range network in particular, was its integration of neurons disconnected from the synaptic connectome. For example, the 14 neuron classes of the pharyngeal nervous system form a heavily synaptically interconnected, functionally autonomous network akin to vertebrate enteric nervous systems, which are topologically isolated from the rest of the nervous system.^70^^,^^79^^,^^80^ Unlike in the wired connectome, there are no neuropeptidergic networks exclusive to the pharynx; to the contrary, pharyngeal neurons are fully integrated into the somatic nervous system via strong reciprocal interconnectivity (Figure 5). All pharyngeal neuron classes receive 90% or more of their incoming connections from outside the pharynx. Some classes of pharyngeal neurons also broadcast extensively to the somatic nervous system, with several (I1, I3, I4, I5, M5, and NSM) having more than 100 outgoing connections (more than 90% of their total) to non-pharyngeal neurons. Likewise, it is notable that the CAN neurons, which completely lack chemical synapses, show strong and reciprocal neuropeptidergic connectivity with the rest of the nervous system, indicating that this unusual neuron class is well embedded in the neural network (Figure 5, CAN highlighted).

**Figure 6:**
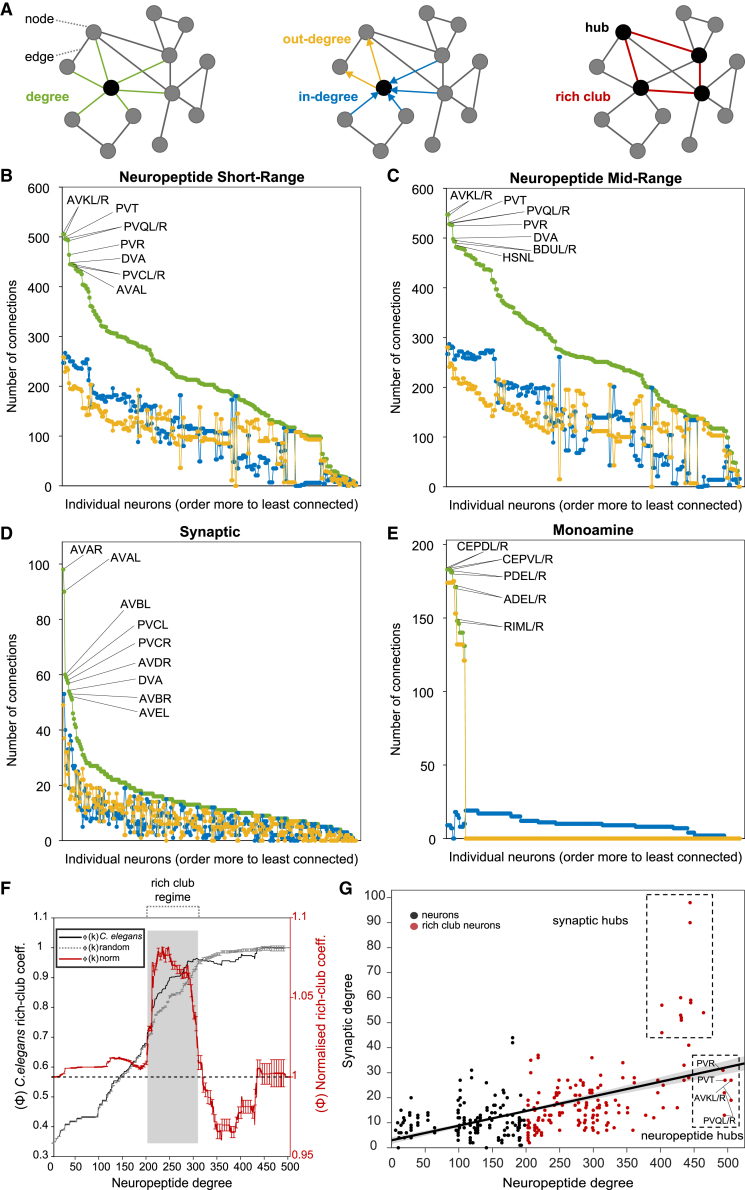
Analysis of peptidergic network degree highlights hubs and a large rich club (A) Network graph representation highlighting nodes (neurons), edges (connections), degree (connection number), hub (highly connected neurons), and rich club (hubs overconnected to each other). (B–E) Degree distributions of *C. elegans* neural networks. In each case, degree (incoming plus outgoing connections) is shown in green, in-degree (incoming connections) in blue and out-degree (outgoing connections) in yellow. The 10 highest-degree hubs in each network are indicated. (F) Rich club analysis. The rich club coefficient Φ(k) for the real *C. elegans* neuropeptidergic network is shown in black; the averaged rich club curves Φ_random_(k) of 100 randomized networks preserving degree distribution is in gray; the red curve is the normalized coefficient (error bars indicate standard deviation). Gray shading indicates the onset of the rich club; for the short-range peptidergic network, this consists of 156 neurons (166 for mid-range, Figure S7). (G) Correlation between synaptic and neuropeptidergic degrees. A positive correlation was observed (r = 0.54, p = 3.1 e^−14^); red dots indicate neurons in the neuropeptidergic rich club; synaptic and peptidergic hubs are highlighted.

We next investigated topological features of the aggregate neuropeptide network. Specifically, we analyzed how peptidergic degree, defined as the number of incoming (in-degree) and outgoing (out-degree) connections, was distributed among neurons. Degree is often an indicator of functional significance in networks, with high-degree nodes (hubs) often playing key functional roles (Figure 6A). As expected from their high density, the average degree of both the short-range (Figure 6B) and mid-range (Figure 6C) networks was significantly higher than for the previously characterized synaptic (Figure 6D) and monoamine (Figure 6E) networks. Moreover, the degree distribution across neuropeptide networks was relatively flat, with many high-degree nodes; more than half the neurons in the short-range network and nearly two-thirds in the mid-range network had a degree higher than 200, indicating in and out connections with at least a third of all other neurons (Figures 6B and 6C). A similarly flat degree distribution was observed in the sparser version of the network based on a 100 nM EC_50_ threshold, indicating that this property is not simply a consequence of high network density (Figure S7). In contrast, the synaptic network was much more centralized, with only 10 neurons having a degree >50 (Figure 6D); similarly, the monoamine network had only 18 high-degree (k > 50) neurons (Figure 6E). The best-connected neurons in the neuropeptide network have exceptionally high in-degree as well as out-degree, indicating that their status as hubs depends as much on incoming connections as on outgoing connections, and as much on their expression of broadly signaling neuropeptides as on integrating GPCRs (Figures 6B and 6C). This contrasts with the monoamine network, where the hubs are exclusively monoamine-releasing neurons with high out-degree (Figure 6E). Interestingly, compared with the synaptic connectome, the neuropeptide connectome has significantly higher clustering and reciprocity (i.e., neurons are more likely to connect in both directions) but lower modularity and disassortativity, further supporting the notion of a decentralized neuropeptide network.

### The neuropeptide connectome contains a rich club and unique peptidergic hubs

A significant feature of many networks is the so-called rich club property. In such networks, the most highly connected hubs form more connections between themselves than expected from their high degree alone; this forms a rich club or core of the network that facilitates communication between more peripheral nodes. The *C. elegans* synaptic connectome, for example, contains a rich club consisting of 11 premotor interneurons that appear to play important roles in driving global brain states.^56^^,^^81^ To determine whether the neuropeptide connectome also contains a rich club, we used a standard computation^82^ to determine whether the network contained a subset of high-degree neurons with a higher degree of interconnection than neurons of equal degree in random graphs. We found that the neuropeptide connectome also shows the rich club property, but its rich club consists of 156 neurons (166 in a mid-range network), more than half of the nervous system (Figure 6F). Within the rich club the density of connections is 0.6834 (p < 0.00001), more than double the density of the overall network (0.3427). A rich club of similar size and composition was observed even if only peptide-receptor interactions with EC_50_ values below 100 nM were considered, indicating that the large rich club was not merely a consequence of high network density (Figure S7). The observation that such a large subset of the nervous system fulfills the topological criteria of a rich club implies that even a very dense and decentralized connectome can be organized to optimize information processing.

To further investigate the relationship between neuropeptide and synaptic signaling, we assessed the correlation between synaptic and neuropeptidergic degree (Figure 6G). We observed that neuropeptide degree and synaptic degree were positively correlated (p < 0.0001, r = 0.53). We further observed that neurons with very high synaptic degree were also neuropeptidergic hubs; for example, all 11 members of the synaptic rich club (DVA, PVCL/R, AVAL/R, AVBL/R, AVDL/R, and AVEL/R) were among the 25 highest-degree nodes in the short-range neuropeptide network (Figure 6F). However, there were also neurons of very high peptidergic degree but unexceptional synaptic degree (Figure 6G). Six neurons (AVKL/R, PVQL/R, PVT, and PVR) had a higher short-range neuropeptide degree than any of the synaptic rich club neurons (Figure 6B); in the mid-range model, these same six neurons remained the highest-degree hubs, followed by the BDU, HSN, and RID neurons (Figure 6C). AVKL/R, PVT, BDUL/R, and RID are notable for expressing no classical neurotransmitters or monoamines,^83^ while the PVQL/R neurons appear anatomically specialized for neuropeptide signaling due to a preponderance of dense-core vesicles.^49^^,^^84^ AVKL/R, RID and PVT have been previously linked to the control of behavioral states related to sleep and arousal,^13^^,^^67^^,^^84^^,^^85^^,^^86^ but the functions of other neuropeptide hub neurons, such as PVQ, PVR, and BDU, are not well characterized. Network analysis thus highlights these neurons as potential targets for future study.

To determine whether the importance of these neuropeptide hubs depends on their expression of particular neuropeptides or receptors, we examined the structures of subnetworks based on specific peptide or receptor classes. Specifically, we examined the networks consisting only of rhodopsin- or secretin-class receptors, networks lacking or containing only RFamide neuropeptides, and networks lacking or containing only promiscuous GPCRs (Figure S8). Interestingly, the neuropeptide hubs from the aggregate network were among the highest-degree hubs in all subdivisions, although the importance of individual neurons varied between subnetworks. In contrast, the synaptic hubs, except DVA, were significantly less important in some network subdivisions; for example, the networks lacking promiscuous receptors or containing only secretin-type receptors. These results further suggest the importance of neuropeptide hubs specialized for peptidergic signaling.

### Mesoscale structure of the neuropeptide connectome core

**Figure 7:**
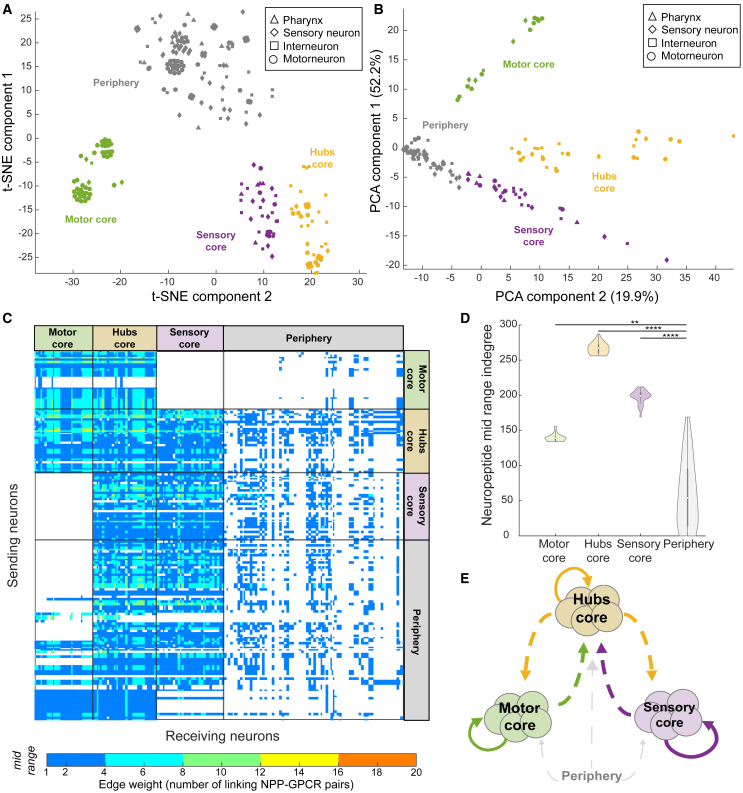
Mesoscale structure of the neuropeptide connectome (A and B) Shown are t-SNE (A) and PCA (B) plots of the adjacency matrix of the mid-range aggregate network (Euclidean distance, perplexity 30). Hubs and core clusters encompassing 112 of 166 neuropeptide rich club neurons, as well as loosely clustered periphery, are indicated by color; datapoint markers represent neuronal classification. (C) Adjacency matrix for the mid-range neuropeptidergic network sorted in both dimensions based on neuronal clusters defined in (A) and (B). (D) Violin plots showing indegree values for the three clusters and the periphery. Median indegree values: motor core, 139; hubs, 267; sensory core, 198; periphery, 54. Indegrees for the four groups were significantly different according to the Kruskal-Wallis test followed by Tukey-Kramer test for multiple comparisons (∗∗p < 0.01; ∗∗∗∗p < 0.0001). (E) Diagram showing connections between clusters. Neurons of the motor core connect with the periphery and with the hubs; the hubs connect to almost every other neuron; the sensory core connects to the hubs and the periphery but not the motor core.

To further probe the structure of the neuropeptide connectome, we investigated whether the network contained modules or other forms of mesoscale substructure. We first applied standard methods for modular decomposition, but the high density of the network precluded the identification of any discrete modules unless we aggressively filtered out lower-weight edges. However, we hypothesized that other types of mesoscale structure may be present.^55^^,^^87^ We wondered, for example, whether we could identify subgroups of neurons with similar peptidergic inputs or outputs, and therefore applied dimensionality reduction methods to the network’s adjacency matrix to identify groups of neurons that shared a connectivity pattern. This analysis highlighted three clearly defined clusters of neurons receiving similar incoming connections, along with a more diffuse cloud of neurons (a.k.a. periphery) with variable connectivity (Figures 7 and S10). Collectively the three clusters comprise 155 neurons, 112 of which (76%) are members of the peptidergic rich club (Figures 7A and 7B; Table S6).

These groups diverge significantly in their indegree (Figure 7D) as well as in the neuron types that form them and to which they are connected (Figures 7C and S10A). Group 1 (a.k.a. motor core) is mostly motor neurons^88^ and its grouping is driven by inputs from mechanosensory neurons PVM and PLML/R^89^ and interneurons PVWL/R (Figure 7C). Group 2 (a.k.a. hubs core), the group with the highest indegree, includes all top neuropeptidergic hubs and receives connections from all neuron types, with neuropeptidergic hubs (PQR, PVT, and PVR) being particularly important drivers (Figure 7C). Finally, group 3 (a.k.a. sensory core) is a mix of neuron types receiving connections from all neuron types except motor neurons, with RIR and pharyngeal neurons I5 and I4 driving the cluster (Figure 7C). Although the characteristic neural inputs of the groups differ substantially, interactions involving versatile neuropeptides FLP-9, FLP-11, and FLP-16 and promiscuous receptors DMSR-1 and DMSR-7 (Figure S10) are important in all clusters and lead to interconnections between groups (Figure 7E). At the neural level, the motor core forms connections with itself, the hubs, and the periphery but not with the sensory core; the sensory core forms connections with the hubs and periphery but not the motor core, and the hubs’ core forms connections with all other groups. Thus, the hubs serve as a link between the sensory and motor cores, which have few direct connections with each other.

### Analysis of receptor and GPCR co-expression

Signaling cascades, in which a neuropeptide receptor is specifically co-expressed with a non-cognate peptide whose release it controls, are a classic hallmark of neuroendocrine pathways. To investigate whether the *C. elegans* neuropeptide connectome contains such cascades, we evaluated co-expression between neuropeptide and receptor genes. We searched for neuropeptide genes and GPCR genes that are co-expressed in the *C. elegans* nervous system more than expected from their individual expression frequencies and identified 121 peptide-receptor pairs meeting this criterion (Figure S11), only 5% of which corresponded to cognate neuropeptide-receptor pairs (autocrine connections). Using these peptide-receptor pairs as nodes and the neuropeptide-receptor interaction data to form edges between them, we built a network of overrepresented signaling cascades within the larger neuropeptide connectome (Figure S11). This network is fully connected and provides a simplified view of how neuropeptide signaling pathways interact within the nervous system, with functional signals organized sequentially despite the dispersed localization of peptides and receptors.

**Figure 8:**
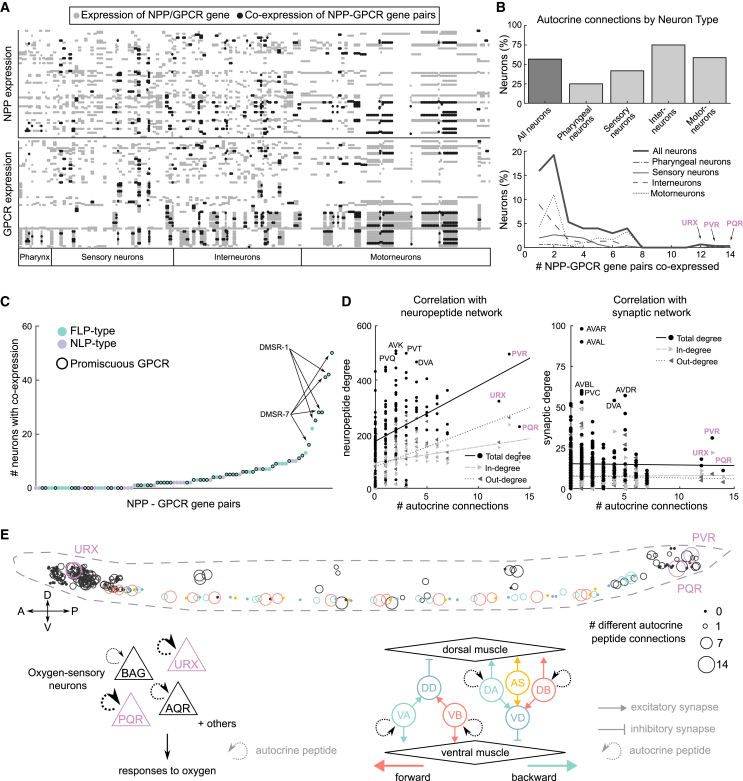
Co-expression between GPCRs and their ligands potentiates autocrine and paracrine signaling (A) Neuronal expression matrix for NPP and GPCR genes of the 92 NPP-GPCR pairs. Gray dots represent expression of only the NPP (upper panel) or GPCR (lower panel), black dots indicate co-expression. (B) Percentage of each neuron type showing peptide autocrine connections (upper panel). The number of different NPP-GPCR pairs co-expressed in each neuron type is shown in the bottom panel. (C) Scatter plot showing the number of neurons with co-expression for each of the 92 NPP-GPCRs. (D) Correlation between number of autocrine connections and neuropeptide (left) or synaptic (right) degree for each neuron. Point shapes indicate degree (round), in-degree (incoming arrow), and out-degree (outgoing arrow). (E) Locations of autocrine connections in the worm. Cell body size indicates the number of autocrine NPP-GPCR pairs expressed in that neuron. Colored neurons (including those of the oxygen-sensing and locomotor circuits, diagrammed below) exhibit the largest number of autocrine connections. Arrow size indicates number of NPP-GPCR pairs.

Co-expression of a neuropeptide receptor with its own ligand will generate a self-loop or autocrine connection, where peptide release can signal back onto the sending cell. We investigated the prevalence of autocrine signaling in the *C. elegans* nervous system, finding that 58% of neurons harbor putative autocrine peptide connections based on ligand-receptor co-expression (Figures 8A and 8B). Autocrine pathways appear most prevalent in interneurons and motor neurons (Figure 8B), although the URX and PQR sensory neurons have among the highest diversity of autocrine signaling. Promiscuous receptors frequently participate in these autocrine loops (Figures 8C and S12), in particular, DMSR-1 and DMSR-7 (Figure 8C). We observed a strong positive correlation between the number of different autocrine peptide connections that a neuron harbors to its degree within the neuropeptide network (Figure 8D), with some but not all neuropeptide hubs showing a high diversity of autocrine signaling. In contrast, weak to no correlations were observed between autocrine diversity and degree in the synaptic, gap junction, or monoamine connectomes (Figures 8D and S12). Autocrine signaling may therefore play a role in regulating the activities of peptidergic hub neurons.

Autocrine connections were especially prevalent in specific circuits in the *C. elegans* nervous system. For example, both URX and PQR are sensory neurons that mediate *C. elegans*’ responses to aversive O_2_ levels^90^^,^^91^^,^^92^; other O_2_ sensory neurons (AQR and BAG) also co-express ligand-receptor pairs, though to a lesser degree (Figure 8E). As O_2_-sensing neurons tonically signal ambient O_2_ concentration,^93^ autocrine signaling may play a stabilizing role, maintaining the homeostasis of neuronal activity. Autocrine neuropeptide signals are also prevalent in the neuromuscular circuit of the ventral nerve cord (VNC), which mediates locomotion.^48^ These autocrine loops are primarily mediated by eight RFamide-related neuropeptides activating two promiscuous receptors, DMSR-1 and DMSR-7, although other receptors such as NPR-5 are also involved (Figure S12). Interestingly, autocrine signaling is restricted to the excitatory A- and B-class motor neurons, which drive backward and forward locomotion, respectively^94^; in contrast, both D-type inhibitory motor neurons and excitatory AS motor neurons, which participate in both forward and backward crawling,^95^ co-express no neuropeptide-GPCR pairs (Figure 8E). Because some autocrine peptidergic pathways are shared between neighboring excitatory motor neurons in the VNC, they might coordinate activity and/or neurosecretion across excitatory motor neurons in a paracrine manner.^88^^,^^96^^,^^97^^,^^98^ Heterogeneity in autocrine pathways within the A- and B-class motor neurons (Figure S12) hints at heterogeneity in the contribution of individual motor neurons in this process. Thus, autocrine/paracrine signaling may act together with synaptic and gap junction connections to control locomotion.

## Discussion

### Neuropeptide signaling forms a complex wireless network

Neuropeptide signaling is critical to brain function, yet despite recent advances in connectomics, the structures of neuropeptidergic signaling networks are largely uncharacterized. We have generated a draft neuropeptide connectome by integrating biochemical data identifying ligands of neuropeptidergic GPCRs,^66^ single-neuron transcriptomic data mapping NPP/GPCR expresssion,^40^ and anatomical data defining neuronal morphologies and contacts. The resulting connectome is remarkably dense; even with highly conservative assumptions about peptidergic diffusion the network is over 10-fold denser than the *C. elegans* synaptic connectome. The conservative assumptions of our models, and the existence of additional still-orphan peptide GPCRs, imply that the actual neuropeptide connectome is even denser than this draft network. However, sensitivity analysis indicates that the overall structure and topology of neuropeptide connectivity is likely to be robust to additional added connections (Figure S7).

A salient feature of the neuropeptide connectome is its decentralized topology, which contrasts sharply with the more centralized structure of wired neural connectomes. Synaptic connectomes from worms to humans are characterized by a core of high-degree hubs, interconnected to form a rich club. This rich club, which in *C. elegans* consists of 11 premotor interneurons, occupies a central position in the connectome, connecting local modules and coordinating their activity. Rich clubs have been linked to important functions; for example, in *C. elegans* the synaptic rich club drives global brain states related to locomotion^56^^,^^81^^,^^99^ and in *Drosophila* it constitutes the main sensorimotor integrative center.^100^ The gap junction and monoamine connectomes of *C. elegans* likewise contain relatively small rich clubs of less than 20 neurons of relatively high degree. In contrast, the neuropeptide connectome contains a rich club of (in the short-range network) 156 neurons, more than half the nervous system. The neurons in this neuropeptidergic rich club are extremely well connected to each other as well as to the rest of the nervous system; based on their degree, each communicates directly with at least 30% of all neurons. This remarkable decentralization may be a feature of neuropeptide signaling networks in other organisms.^43^^,^^101^ The finding that even such highly decentralized networks show rich club structure implies that their structure is nonetheless optimized for efficient communication.

### Computational implications of neuropeptide network structure

The decentralized structure of the neuropeptidergic connectome implies that it may employ different strategies for computation and information flow than more centrally organized synaptic networks. Intriguingly, both nematode and mammalian neuronal types appear to express nearly unique combinations of neuropeptides and receptors, potentially serving as a molecular bar code for neural identity.^40^^,^^43^ Thus, the source of a signal may be encoded by the precise combination of peptides released by sending neurons. The dynamics of neuropeptide release may also influence the information conveyed by peptidergic signals. For example, acute release of FLP-20 peptides by mechanosensory neurons triggers short-term sensory and locomotor arousal in response to touch stimulation,^14^ while chronic release of FLP-20 peptides from the same neurons mediates long-term cross-modal plasticity in olfactory circuits in the absence of touch sensing.^102^ Future studies of such mechanisms may provide general insights into how neuropeptide networks encode information in the brain.

Despite its dense connectivity, the core of the neuropeptide connectome exhibits a clear substructure. Analysis of peptidergic inputs clustered neurons in the network core into three clear groups: a sensory core, a motor core, and a group centered around the main hubs. These three groups themselves connect in a defined pattern, with the hubs linking the sensory and motor cores, which show few direct links with each other. This organization contrasts in interesting ways with the organization of many synaptic networks, where peripheral neurons form modules with high internal connectivity that connect to each other through the hubs of the rich club core. In the neuropeptide connectome the core itself exhibits a clear mesoscale organization, with its three subgroups defined not by unusually high intra-group connectivity but by intra-group similarity of incoming and outgoing connection patterns. This parallels recent work showing the existence of different types of mesoscale organization^87^ and is also reminiscent of stochastic block modeling approaches previously applied to the *C. elegans* synaptic connectome.^55^ It will be interesting to see whether neuromodulatory and synaptic networks from other nervous systems show a similar diversity of meso-scale structure, encompassing both classical modules as well as classes of neurons that are not interconnected but have similar connectivity profiles and perform similar functions.

### Identification of neuropeptide signaling hubs

Although the rich club of the neuropeptide connectome is extensive and encompasses a large portion of the *C. elegans* nervous system, some of its neurons exhibit a particularly high degree and may play key roles in neuromodulatory signaling. Not surprisingly, the neurons of the synaptic rich club are highly connected through neuropeptides; because these neurons play important roles driving global brain states,^81^^,^^99^^,^^103^ it is logical that they would also be important targets of neuromodulatory regulation. The neuropeptide connectome also contains neurons whose neuropeptide degree is higher than any of the synaptic rich club. Two classes (PVT and AVK) are specialized peptidergic neurons expressing no classical neurotransmitter or monoamine, which have been linked to arousal and sleep-like behaviors.^13^^,^^84^^,^^85^^,^^104^^,^^105^ Specialized neuropeptidergic neurons have also been described in other organisms, in some cases linked to global behaviors such as fear.^106^ Other peptidergic hubs (PVR and PVQ) are tail neurons that extend long processes to the nerve ring and, in the case of the PVQs and PVT, these processes are rich in dense-core vesicles.^49^^,^^71^ Because AVK also has an unusually long process, peptidergic hubs may be morphologically specialized for the local release of peptides throughout the nervous system. With the notable exception of AVK,^13^^,^^86^ the functions of most nematode neuropeptide hubs are uncharacterized. Given their importance in the neuropeptide network, it will be interesting in the future to explore the roles of these neurons in the control of behavioral states.

Strikingly, even in the long-range network, which imposes no spatial restrictions on neuropeptide diffusion, the top short- and mid-range hub neurons retain their central importance in the network. This indicates that their high degree is not merely an artifact of the diffusion model we imposed but rather results from expressing key combinations of neuropeptides and receptors. Notably, the long-range network contains additional high-degree nodes that are not hubs in the short- and mid-range networks (e.g., the oxygen sensors URX, AQR, and PQR and motor neurons DA09 and VA12), which could potentially engage in long-range neuroendocrine signaling. Conversely, the long-process morphology of the short- and mid-range hubs might allow them to carry out global neuromodulation on a finer temporal or spatial scale. In the future, these questions may be addressable using *in vivo* probes for neuropeptide-receptor signaling.^107^^,^^108^

### Neuropeptide signaling links nervous system components

In addition to hubs, the neuropeptide connectome also contains edges of unusually high weight, representing neuron pairs linked by multiple neuropeptide-signaling pathways. In particular, 17 pairs of neurons, principally in the oxygen-sensing circuit, are linked by 15 or more different neuropeptide-receptor couples. Why might oxygen-sensing neurons participate in so many complex neuromodulatory interactions? These neurons strongly influence locomotor states, and their tonic responses to ambient oxygen are influenced by experience and other sensory cues.^90^^,^^91^^,^^92^ Complex neuropeptide signaling may allow feedback between the oxygen sensors and motor neurons to fine-tune the activity of this circuit across time and space. Understanding how neurons can process complex, parallel neuropeptidergic signals may provide insight into similar processes in larger brains.

The oxygen-sensing neurons and the motor circuit are also important sites for autocrine signaling in which a neuropeptide receptor and its ligand are co-expressed in the same neuron. Autocrine peptide signaling is also ubiquitous in other brains^44^^,^^109^ and may support cell-autonomous feedback to maintain neuronal homeostasis,^68^^,^^110^^,^^111^^,^^112^^,^^113^^,^^114^ potentially important in the tonically signaling oxygen-sensing neurons. In the motor circuit, ostensibly autocrine signaling may coordinate the physiology of neighboring neurons with similar gene expression,^115^^,^^116^ modulating the proprioceptive and electrical coupling that generates waves of muscle contraction over adjacent body regions.^88^ Indeed, mutants for several neuropeptides acting in motor neuron autocrine pathways have been shown to have locomotion defects,^117^^,^^118^ suggesting an important role in patterning locomotor behavior.

Neuropeptide signaling also plays a critical role in linking disconnected components of the nervous system to the broader network. In particular, the pharyngeal neurons, which form a wired network analogous to the vertebrate's enteric nervous system, and the CAN neurons, with processes in the canal-associated nerve, are virtually unconnected to the synaptic and gap junction connectomes yet are extensively integrated into the neuropeptide connectome. Both CAN and the pharyngeal neurons express multiple GPCRs, whose ligands are expressed exclusively outside the pharynx or canal-associated nerve, indicating that these neurons are regulated by peptides released from physically unconnected processes. Despite their disconnection from the wired connectomes, the pharynx and CAN neurons carry out essential physiological functions; indeed, CAN and the pharyngeal neuron M4 are the only neurons whose ablation is lethal to the animal.^119^^,^^120^ Peptidergic signaling thus provides a means for communication between the wider nervous system and these isolated but biologically critical neurons. Understanding the functions of these neurons could shed light on neurons with few synaptic partners in other organisms, such as *Drosophila* and *Platynereis*.^101^^,^^121^

### Prospects for mapping wireless brain connectomes

We describe here the draft neuropeptide connectome of *C. elegans*. In the future, we plan to refine this connectome, for example, by deorphanization of neuropeptide receptors with currently unknown ligands. Differential posttranscriptional processing may also generate different peptides and receptors in neurons expressing the same gene, and alternative patterns of gene expression during development or in response to environmental cues may alter the structure and function of neuropeptide signaling networks. Moreover, non-neuronal cells function as both senders and receivers of neuropeptide signals; with the use of reporter lines for peptides and receptors, it should be possible to incorporate these cells into the neuropeptide connectome. Finally, *in vitro* experiments could identify the G protein pathways downstream of individual receptors and *in vivo* sensors could provide empirical data on the spatial scope of neuropeptide signaling pathways. Together, these data will facilitate functional modeling of neuropeptidergic circuits revealed in the connectome maps.

In principle, the approaches described here should also be applicable to mapping the peptidergic networks of animals with larger brains. Transcriptomic data from various vertebrates indicates that, as in *C. elegans*, most neurons express multiple neuropeptides and GPCRs, facilitating dense and potentially decentralized networks.^18^^,^^39^^,^^43^ Although it is currently not possible to precisely link gene expression clusters with individual neurons in most animals, targeted reporters should eventually make it possible to relate neuropeptide and receptor expression to increasingly detailed connectome maps in flies and mice. Basic mechanisms of neuropeptide signaling are shared in all animals, from nematodes to mammals, and although the *C. elegans* nervous system is anatomically small, at the molecular level its neuropeptide systems are highly complex and show significant homology to other animals. Thus, the neuropeptide connectome of *C. elegans* may serve as a prototype to unravel general principles of neuromodulatory network structure that also apply to much larger brains.

## STAR★Methods

### Key resources table

**Table:** 

REAGENT or RESOURCE	SOURCE	IDENTIFIER
**Experimental models: Organisms/strains**		

*flp-1(syb2658[flp-1::T2A::3XNLS::GFP]) IV*	Taylor et al.^40^	PHX2658
*flp-14(syb3323[flp-14::T2A::3xNLS::GFP]) III*	Sun and Hobert^123^	PHX3323
*nlp-13(syb3411[nlp-13::T2A::3xNLS::GFP]) V*	Sun and Hobert^123^	PHX3411
*nlp-58(syb3191 [nlp-58::T2A::3xNLS::GFP]) V*	Sun and Hobert^123^	PHX3191
*nlp-45(ot1032[nlp-45::T2A::GFP::H2B]) X*	This paper	OH16380
*flp-26(syb3588[flp-26::T2A::3xNLS::GFP]) X*	Sun and Hobert^123^	PHX3588
*ins-9(syb2616[ins-9::T2A::3xNLS::GFP]) X*	Sun and Hobert^123^	PHX2616
*ins-9(syb5536[ins-9::SL2::gfp::H2B]) X*	Sun and Hobert^123^	PHX5536
*flp-28(syb3207[flp-28::T2A::3xNLS::GFP]) X*	This paper	PHX3207
*flp-3(syb2634[flp-3::T2A::3XNLS::GFP]) X*	Sun and Hobert^123^	PHX2634
*ins-6(syb2685[ins-6::T2A::3xNLS::GFP]) II*	Tekieli et al.^124^	PHX2685
*ins-6(syb5463[ins-6::SL2::GFP::H2B]) II; otIs669 him-5(e1490) V*	Reilly et al.^125^	OH17767
*nlp-50(syb2704[nlp-50::T2A::3xNLS::GFP]) II*	Sun and Hobert^123^	PHX2704
*flp-20 (syb3241 [flp-20::T2A::3xNLS::GFP]) X*	This paper	PHX3241
*flp-20(syb4049[flp-20::SL2::GFP::H2B]) X*	This paper	PHX4049
*nlp-51(syb2805 [nlp-51::T2A::3xNLS::GFP]) II*	Taylor et al.^40^	PHX2805
*nlp-51(syb3936[nlp-51::SL2::GFP::H2B]) II*	Reilly et al.^125^	PHX3936
*trh-1(syb4421[trh-1::SL2::GFP::H2B]) IV*	Vidal et al.^80^	PHX4421
*flp-5(syb4513[flp-5::SL2::GFP::H2B]) X*	Vidal et al.^80^	PHX4513
*flp-32 (T2A::3xNLS::GFP)*	This paper	N/A
*flp-32(syb4374[flp-32::SL2::gfp::H2B]) X*	Cros and Hobert^126^	PHX4374
*flp-27 (T2A::3xNLS::GFP)*	Tekieli et al.^124^	N/A
*flp-27(syb4413 [flp-27::SL2::GFP::H2B]) II*	Reilly et al.^125^	PHX4413
*npr-37(syb4440[npr-37::SL2::GFP::H2B]) IV*	Cros and Hobert^126^	PHX4440
*dmsr-2(syb4514 [dmsr-2::SL2::gfp::H2B]) I*	Cros and Hobert^126^	PHX4514
*dmsr-6 (syb4442 [dmsr-6::SL2::GFP::H2B]) I*	This paper	PHX4442
*npr-32 (syb4433 [npr-32::SL2::GFP::H2B] IV*	This paper	PHX4433
*aex-2(syb4447 [aex-2::SL2::GFP::H2B)] X*	Cros and Hobert^126^	PHX4447
*trhr-1(syb4453[trhr-1::SL2::GFP::H2B]) I*	Vidal et al.^80^	PHX4453
*trk-1 (SL2::GFP::H2B)*	This paper	N/A
*frpr-19(syb4523[frpr19::SL2::GFP::H2B]) IV*	This paper	PHX4523
*nmur-2(syb4517 [nmur-2::SL2::GFP::H2B)] II*	This paper	PHX4517

**Software and algorithms**		

MATLAB	MathWorks	R2023a
Brain Connectivity Toolbox	Rubinov et al.^127^	v2019-03-03
Neuropeptide Networks Analysis	This paper	https://github.com/LidiaRipollSanchez/Neuropeptide-Connectomehttps://zenodo.org/badge/latestdoi/560503626https://doi.org/10.5281/zenodo.8387059
Cytoscape	Shannon et al.^128^	https://cytoscape.org
MuxViz	De Domenico et al.^129^	https://github.com/manlius/muxViz
NeuroPAL Automated Cell ID	Yemini et al.^69^	https://github.com/amin-nejat/CELL_ID
CeNGEN	Taylor et al.^40^	https://www.cengen.org
NemaNode	Witvliet et al.^71^	https://www.nemanode.org
NemaMod	This paper	https://www.nemamod.org.

**Other**		

Confocal Laser Scanning Microscope	Zeiss	LSM 880

### Resource availability

#### Lead contact

Further information and requests for resources and strains should be directed to and will be fulfilled by the lead contact, William Schafer (wschafer@mrc-lmb.cam.ac.uk).

#### Materials availability

Strains generated in this study have been deposited in the CGC.

### Experimental model and study participant details

#### Worms and maintenance

Wild type *Caenorhabditis elegans* were Bristol strain N2. All *C. elegans* were young-adult hermaphrodites. All strains were raised at 20^o^C, on nematode growth media (NGM) plates, and fed OP50 *Escherichia coli* as previously described.^122^

### Method details

#### Reporter transgenic strains

Thirty-two transcriptional C-terminal GFP reporters of neuropeptides and neuropeptide receptors were created by CRISPR-Cas9 genome engineering. GFP was inserted in the last coding exon of the gene of interest using different cassettes. Of the 17 total neuropeptide precursor genes that we made reporters for, 8 had only a T2A::3xNLS::GFP tag, 1 had a T2A::GFP::H2B tag, 6 had both T2A::3xNLS::GFP and SL2::GFP::H2B tags, and 2 had only an SL2::GFP::H2B tag (Table S1). For the GPCR receptors, 8 gene expression reporters were made all with SL2::GFP::H2B tags (Table S2).

Transgenic strains used in this study are available in the supplement (Tables S1 and S2) and key resources table.^80^^,^^125^^,^^126^^,^^124^^,^^123^ Most of these strains were made by SunyBiotech.

#### Reporter analysis

Young adult animals were mounted on 5% agarose pads and immobilized with 100 mM sodium azide and imaged on a Zeiss LSM880 using a 40X objective lens. GFP expression reporters were identified at single neuron resolution as described.^69^ GFP reporter expression of these constructs, as reported in Tables S1 and S2 were noted using three categories: moderate to high expression, low and variable expression, and no detected expression. Additionally, we compared our GFP reporter expression data to single-cell RNA-seq expression (scRNAseq) data from the CeNGEN project using their standard thresholds (4 being the most stringent, 1 being the least stringent, and blank be unfiltered).^40^

In our analysis, for each gene and each CeNGEN threshold, we tallied 1) the number of neurons that showed GFP expression but not scRNA expression, 2) the number of neurons showing both GFP expression and scRNA expression, and 3) the number of neurons that showed scRNA expression but no GFP expression (Table S3). Based on the results of this analysis threshold 4, although in some occasions too conservative, had the best correlation between GFP reporter and CeNGEN scRNAseq data expression per neuron for the tested NPP and GPCR genes.

#### Synaptic and gap junction networks

The synaptic and gap junction networks used in this work were based on the full hermaphrodite *C. elegans* connectome, containing all 302 neurons. This network was composed from the somatic connectome,^49^ updated and released by the Chklovskii lab^47^; and the pharyngeal network of Albertson and Thomson,^70^ made available by the Cybernetic *Caenorhabditis elegans* Program (CCeP). The functional classifications referred to in the text (i.e., *sensory neuron*, *interneuron*, *motor neuron*) are based on the classification scheme used in WormAtlas.^130^ When there is double or triple classification in nerve ring neurons that can be sensory neurons, interneurons, or motor neurons, the Zhen lab classification^71^ was used to select one neuron type. URBL and URBR are the only neurons in which the WormAtlas and the Zhen lab classification diverge, leading us to classify them as sensory neuron following the later most recent classification. DB neurons are identified as motor neurons although WormAtlas indicates that these could also be interneurons.^130^ The gap junction network was modelled as an undirected network with bidirectional electrical synapses; note however that some gap junctions might be rectifying and thus exhibit directionality. In the synaptic network reciprocal connections between nodes are considered as two separate unidirectional connections.

#### Monoamine network construction

The monoamine network used in this work was made following the same procedure by Bentley et al.^45^ The monoamine expression for the 302 neurons comes from the neurotransmitter atlas of *C. elegans*^83^ and receptor expression for the 302 neurons comes from the single-cell expression data from the CeNGEN project (https://www.cengen.org).^40^ We used the expression data at CeNGEN threshold 4. The interactions between ligand and receptors were previously described.^45^ The adjacency matrix was built using a binary version of the expression data for the 302 neurons. For a given point A^*M*^(*i*,*j*) and for a given monoamine receptor pair *M* the connection between two neurons is defined by A^*M*^(*i*,*j*) = Mon^*M*^(*i*,*j*) × Receptor^*M*^(*i*,*j*). Each monoamine receptor interaction forms an individual binary network. To get the overall monoamine network we add each individual monoamine receptor network resulting in a weighted network where the weight indicates the number of monoamine receptor pairs that connect two nodes. Reciprocal connections between nodes are considered as two separate unidirectional connections.

#### Neuropeptide network construction

The neuropeptide network used in this work was made using a similar approach to that used for the monoamines. The interactions between ligands and receptors were identified using a large-scale *in vitro* reverse pharmacology pipeline in which over 87% of the predicted peptide GPCRs were challenged with FMRFamide related peptides (FLP) and non-insulin non-FLP like peptides (NLP).^131^ Neuropeptide precursor and GPCR gene expression for the 302 neurons was extracted from the single-cell transcriptome data of the CeNGEN project (https://www.cengen.org).^40^ We used the expression data at CeNGEN threshold 4. The adjacency matrix was built using a binary version of the expression data for the 302 neurons. For a given point A^*N*^(*i*,*j*) and for a given neuropeptide receptor pair *N* the connection between two neurons is defined by A^*N*^(*i*,*j*) = NPP^*N*^(*i*,*j*) × GPCR^*N*^(*i*,*j*). Each neuropeptide receptor interaction forms an individual binary network. To get the overall neuropeptide network we add each individual neuropeptide receptor network resulting in a weighted network where the weight indicates the number of neuropeptide receptor pairs that connect two nodes. Reciprocal connections between nodes are considered as two separate unidirectional connections. Details and salient features of all networks are summarized in Table S5 and at https://github.com/LidiaRipollSanchez/Neuropeptide-Connectome.

#### Neuropeptide network spatial constraining

Neuropeptidergic networks were locally thresholded to filter out connections between neurons that were anatomically far from each other. The anatomical EM data was obtained from The Mind of the Worm (https://www.wormatlas.org/MoW_built0.92/MoW.html) and other literature.^49^^,^^70^^,^^71^ These data were used to create a table of locations for each neuronal process, identifying 27 different neuronal process bundles in the *C. elegans* nervous system as previously defined.^49^ This classification was then used to filter out neuropeptidergic connections based on putative signaling ranges. The stringent short-range thresholding allows connections only between neuronal processes that are in the same process bundle and the pharynx is a separated system where connections are allowed between pharyngeal neurons only. The mid-range stringency thresholding allows connections between neurons with neuronal processes in the same anatomical area: head (including pharynx and the ventral cord neurons that are in the ventral ganglion), midbody and tail. In the long-range (unthresholded) system all neuropeptidergic connections are allowed.

#### Topological network measures

Edge counts, adjacency matrices and reducibility clusters were all computed using binary directed versions of the networks. The same networks, excluding self-connections (i.e., setting all diagonal elements to 0), were used to compute all other measures.

Network measures are compared to 100 null model networks generated using the degree-preserving edge swap procedure from the Brain Connectivity Toolbox for MATLAB.^127^ This is performed by selecting a pair of edges (*A*→*B*) (*C*→*D*) and swapping them to give (*A*→*D*) (*C*→*B*). If the resulting edges already exist in the network, another pair of edges is selected instead. Each edge was swapped 10 times to ensure full randomization.

#### Degree

Degree is the number of edges connected to a given node. Indegree is the number of incoming connections connected to a given node and outdegree is the number of outgoing connections.

#### Density

Density *d* is the fraction of present connections *K* to possible connections between the given nodes *N*: $d=\frac{K}{N^{2}-N}$

#### Clustering coefficient

Transitivity defines the ratio of triangles to triples in the network (where a triple is a single node with edges running to an unordered pair of others, and a triangle is a fully connected triple). For a directed network, this is equivalent to: $T=\frac{\sum_{i\in N}t_{i}}{\sum_{i\in N}\left[\left(k_{i}^{out}+k_{i}^{in}\right)\left(k_{i}^{out}+k_{i}^{in}\right)-10-2\sum_{j\epsilon N}A_{ij}A_{ji}\right]}$ where *A* is the adjacency matrix, *N* is the number of nodes, *k*^out^ and *k*^in^ are the out-degree and in-degree, and *t*_*i*_ is the number of triangles around a node: $t_{ij}=\frac{1}{2}\sum_{j,h\epsilon N}(A_{ij}+A_{ji})\left(A_{ih}+A_{hi}\right)\left(A_{jh}+A_{hj}\right)$

#### Reciprocity

Reciprocity is the fraction of reciprocal edges in the network: $r=\frac{\left|E^{↔}\right|}{M}$where *M* is the number of edges, and |*E*^↔^| is the number of reciprocal edges: $\left|E^{↔}\right|=\sum_{i\neq j}A_{ij}A_{ji}$

#### Rich club coefficient

The rich club coefficient measures the tendency for high-degree nodes in a network to form highly interconnected communities.^56^ These communities can be identified by creating subnetworks for each degree level *k* and removing nodes with a degree ≤ *k*. Then the rich club coefficient Φ(*k*) for each subnetwork is defined as the ratio of connections in the subnetwork *M*_*k*_ to the number of maximum possible connections. For a directed network with no self-connections, where *N*_*k*_ is the number of remaining nodes, this is given by:$\phi_{\left(k\right)}=\frac{M_{k}}{N_{k}\left(N_{k}-1\right)}$

Thus, a fully connected subnetwork at a given degree *k* has a rich club coefficient Φ(*k*) = 1. We normalize the rich-club coefficient by calculating:$\phi_{norm\left(k\right)}=\frac{\phi \left(k\right)}{\langle \phi_{random}\left(k\right)\rangle }$ where ⟨Φ_*random*_(k)⟩ is the average value of the rich club coefficient across random networks.

A rich club exists when Φ_*norm*_(*k*) ≥ 1, but in order to get a clear threshold range we use a probabilistic approach. The threshold range of the rich club is defined by Φ_*norm*_(*k*) ≥ 1 + 1*σ*, where *σ* is the Standard Deviation of Φ_*random*_(*k*) for the 100 random networks.

#### Dimensionality reduction analysis

t-SNE is an algorithm for dimensionality reduction that facilitates visualizing high dimensionality data. The analysis described here was performed using the MATLAB *t-sne* function on the adjacency network of connections. The neuropeptide dimension was reduced, and clustering was performed based on the pattern of connections due to receptor expression. Different distance measures were tested to confirm the clustering: Euclidean distance, Chebychev distance, cosine distance and Mahalanobis distance.

#### Co-expression analysis

The signaling networks used in this work represent connections between co-occurring genes. Nodes are defined as pairs of neuropeptide precursor and GPCR genes that co-occur more than expected by chance as measured by a Fisher’s exact test^132^ with FDR (false positive rate) correction.^133^ The 2×2 contingency table for the Fisher’s exact test contains the number of neurons for which both genes co-occur and the number of neurons in which each NPP and GPCR gene is expressed without co- occurring with the second gene. Thus, the Fisher’s test is defined as:

$$p=\frac{\left(R_{1}!R_{2}!\right)\left(C_{1}!C_{2}!\right)}{N!\prod_{i,j}n_{i,j}!}$$

Where R_1_ and R_2_ are the row sums, C_1_ and C_2_ are the column sums, *N* is the total number of observations in the contingency table, and n_ij_ is the value in the *i*th row and *j*th column of the table

Interactions between nodes are defined by the receptor-ligand interactions that the co-occurring genes have with genes that co-occur in another node. The interactions between ligand and receptors were identified using a large-scale *in vitro* reverse pharmacology pipeline.^133^

#### Software used

Network measures were computed in MATLAB (v9.8.0.1323502 (R2020a), The MathWorks Inc., Natick, MA) using the Brain Connectivity Toolbox^127^ (v2019-03-03) and the MATLAB/Octave Networks Toolbox.^134^ Clustering and visualization of multilayer plots was performed using MuxViz.^129^ Additional network visualizations were created using Cytoscape.^128^ Figures were composed and edited using Adobe Illustrator (2023) and Adobe Acrobat (2023). Worm anatomy drawings were made using Adobe Illustrator (2023). The website was built in ShinyApps.

### Quantification and statistical analysis

#### Statistical analysis

Statistical details of the analysis done can be found in the following section. Statistical data is reported in the main text, figures, and tables as noted. Significance adheres to the common standard, after adjusting for multiple testing, of p % 0.05. The symbols ^∗^, ^∗∗^, ^∗∗∗^, and ^∗∗∗∗^ refer to p % 0.05, 0.01, 0.001, and 0.0001, respectively. Not significant is described as n.s. The n for each statistical test is described in each figure.

When error bars are presented in the figures they represent the standard deviation.

#### Statistical analysis software

Statistical analysis was performed using MATLAB (v9.8.0.1323502 (R2023a), The MathWorks Inc., Natick, MA) using the Statistics and Machine Learning Toolbox (v12.5).

### Additional resources

All data can be accessed and interacted with in the project website https://www.nemamod.org. Code and data can be accessed in https://github.com/LidiaRipollSanchez/Neuropeptide-Connectome.

## Data Availability

Data have been deposited at Figshare and are publicly available as of the date of publication (https://doi.org/10.6084/m9.figshare.c.6895870.v1). DOIs are listed in the key resources table. All original code has been deposited at https://github.com/LidiaRipollSanchez/Neuropeptide-Connectome and is publicly available as of the date of publication.

## Supplemental information

Document S1. Figures S1–S12

Table S1. Reporter expression analysis for neuropeptide precursor genes, related to Figure 2

Table S2. Reporter expression analysis for GPCR genes, related to Figure 2

Table S3. Comparison between reporter and RNA-seq expression analyses, related to Figure 2

Table S4. Anatomical positions of neurons and processes, related to Figure 3

Table S5. Summary of individual peptide-receptor couples and their networks, related to Figure 4

Table S6. Summary of individual neuron properties, related to Figure 7

Document S2. Article plus supplemental information

## References

[1] Scheffer L.K., Xu C.S., Januszewski M., Lu Z., Takemura S.Y., Hayworth K.J., Huang G.B., Shinomiya K., Maitlin-Shepard J., Berg S. (2020). A connectome and analysis of the adult Drosophila central brain. eLife.

[2] Csaba V., Sanja J., Martin G., Réza S., Nobuo U., James David B., Sara M., Konrad H., Luis Alberto B.-C., Elizabeth W. (2020). Whole-animal connectome and cell-type complement of the three-segmented Platynereis dumerilii larva. bioRxiv.

[3] Ryan K., Lu Z., Meinertzhagen I.A. (2016). The CNS connectome of a tadpole larva of Ciona intestinalis (L.) highlights sidedness in the brain of a chordate sibling. eLife.

[4] Kunst M., Laurell E., Mokayes N., Kramer A., Kubo F., Fernandes A.M., Förster D., Dal Maschio M., Baier H. (2019). A cellular-resolution atlas of the larval zebrafish brain. Neuron.

[5] Oh S.W., Harris J.A., Ng L., Winslow B., Cain N., Mihalas S., Wang Q., Lau C., Kuan L., Henry A.M. (2014). A mesoscale connectome of the mouse brain. Nature.

[6] Eichler K., Li F., Litwin-Kumar A., Park Y., Andrade I., Schneider-Mizell C.M., Saumweber T., Huser A., Eschbach C., Gerber B. (2017). The complete connectome of a learning and memory centre in an insect brain. Nature.

[7] Hulse B.K., Haberkern H., Franconville R., Turner-Evans D., Takemura S.Y., Wolff T., Noorman M., Dreher M., Dan C., Parekh R. (2021). A connectome of the Drosophila central complex reveals network motifs suitable for flexible navigation and context-dependent action selection. eLife.

[8] Nässel D.R. (2009). Neuropeptide signaling near and far: how localized and timed is the action of neuropeptides in brain circuits?. Invert. Neurosci..

[9] van den Pol A.N. (2012). Neuropeptide transmission in brain circuits. Neuron.

[10] Atkinson L.E., Liu Y., McKay F., Vandewyer E., Viau C., Irvine A., Rosa B.A., Li Z., Liang Q., Marks N.J. (2021). Ascaris suum Informs extrasynaptic volume transmission in nematodes. ACS Chem. Neurosci..

[11] Nurrish S. (2014). Dense core vesicle release: controlling the where as well as the when. Genetics.

[12] Marder E. (2012). Neuromodulation of neuronal circuits: back to the future. Neuron.

[13] Oranth A., Schultheis C., Tolstenkov O., Erbguth K., Nagpal J., Hain D., Brauner M., Wabnig S., Steuer Costa W., McWhirter R.D. (2018). Food sensation modulates locomotion by dopamine and neuropeptide signaling in a distributed neuronal network. Neuron.

[14] Chew Y.L., Tanizawa Y., Cho Y., Zhao B., Yu A.J., Ardiel E.L., Rabinowitch I., Bai J., Rankin C.H., Lu H. (2018). An afferent neuropeptide system transmits mechanosensory signals triggering sensitization and arousal in C. elegans. Neuron.

[15] Lin S., Senapati B., Tsao C.H. (2019). Neural basis of hunger-driven behaviour in Drosophila. Open Biol..

[16] Ramachandran S., Banerjee N., Bhattacharya R., Lemons M.L., Florman J., Lambert C.M., Touroutine D., Alexander K., Schoofs L., Alkema M.J. (2021). A conserved neuropeptide system links head and body motor circuits to enable adaptive behavior. eLife.

[17] Melzer S., Newmark E.R., Mizuno G.O., Hyun M., Philson A.C., Quiroli E., Righetti B., Gregory M.R., Huang K.W., Levasseur J. (2021). Bombesin-like peptide recruits disinhibitory cortical circuits and enhances fear memories. Cell.

[18] Zhong W., Barde S., Mitsios N., Adori C., Oksvold P., Feilitzen K.V., O'Leary L., Csiba L., Hortobágyi T., Szocsics P. (2022). The neuropeptide landscape of human prefrontal cortex. Proc. Natl. Acad. Sci. USA.

[19] Bargmann C.I. (2012). Beyond the connectome: how neuromodulators shape neural circuits. Bioessays.

[20] Schoofs L., De Loof A., Van Hiel M.B. (2017). Neuropeptides as regulators of behavior in insects. Annu. Rev. Entomol..

[21] Bhat U.S., Shahi N., Surendran S., Babu K. (2021). Neuropeptides and behaviors: how small peptides regulate nervous system function and behavioral outputs. Front. Mol. Neurosci..

[22] Yañez-Guerra L.A., Thiel D., Jékely G. (2022). Premetazoan origin of neuropeptide signaling. Mol. Biol. Evol..

[23] Jékely G. (2021). The chemical brain hypothesis for the origin of nervous systems. Philos. Trans. R. Soc. Lond. B Biol. Sci..

[24] Van Bael S., Watteyne J., Boonen K., De Haes W., Menschaert G., Ringstad N., Horvitz H.R., Schoofs L., Husson S.J., Temmerman L. (2018). Mass spectrometric evidence for neuropeptide-amidating enzymes in Caenorhabditis elegans. J. Biol. Chem..

[25] Frooninckx L., Van Rompay L., Temmerman L., Van Sinay E., Beets I., Janssen T., Husson S.J., Schoofs L. (2012). Neuropeptide GPCRs in C. elegans. Front. Endocrinol. (Lausanne).

[26] Jékely G. (2013). Global view of the evolution and diversity of metazoan neuropeptide signaling. Proc. Natl. Acad. Sci. USA.

[27] Rizzo M.J., Johnson E.C. (2020). Homodimerization of Drosophila Class A neuropeptide GPCRs: evidence for conservation of GPCR dimerization throughout metazoan evolution. Biochem. Biophys. Res. Commun..

[28] Rogers C., Reale V., Kim K., Chatwin H., Li C., Evans P., de Bono M. (2003). Inhibition of Caenorhabditis elegans social feeding by fmrfamide-related peptide activation of NPR-1. Nat. Neurosci..

[29] Beets I., Janssen T., Meelkop E., Temmerman L., Suetens N., Rademakers S., Jansen G., Schoofs L. (2012). Vasopressin/oxytocin-related signaling regulates gustatory associative learning in C. elegans. Science.

[30] Iannacone M.J., Beets I., Lopes L.E., Churgin M.A., Fang-Yen C., Nelson M.D., Schoofs L., Raizen D.M. (2017). The RFamide receptor DMSR-1 regulates stress-induced sleep in C. elegans. eLife.

[31] Nässel D.R., Zandawala M. (2019). Recent advances in neuropeptide signaling in Drosophila, from genes to physiology and behavior. Prog. Neurobiol..

[32] Watteyne J., Peymen K., Van der Auwera P., Borghgraef C., Vandewyer E., Van Damme S., Rutten I., Lammertyn J., Jelier R., Schoofs L. (2020). Neuromedin U signaling regulates retrieval of learned salt avoidance in a C. elegans gustatory circuit. Nat. Commun..

[33] Topilko T., Diaz S.L., Pacheco C.M., Verny F., Rousseau C.V., Kirst C., Deleuze C., Gaspar P., Renier N. (2022). Edinger-Westphal peptidergic neurons enable maternal preparatory nesting. Neuron.

[34] Davenport A.P., Scully C.C.G., de Graaf C., Brown A.J.H., Maguire J.J. (2020). Advances in therapeutic peptides targeting G protein-coupled receptors. Nat. Rev. Drug Discov..

[35] Coleman P.J., Gotter A.L., Herring W.J., Winrow C.J., Renger J.J. (2017). The discovery of suvorexant, the first orexin receptor drug for insomnia. Annu. Rev. Pharmacol. Toxicol..

[36] Hargreaves R., Ferreira J.C., Hughes D., Brands J., Hale J., Mattson B., Mills S. (2011). Development of aprepitant, the first neurokinin-1 receptor antagonist for the prevention of chemotherapy-induced nausea and vomiting. Ann. N. Y. Acad. Sci..

[37] Zhang Z.Q., Hölscher C. (2020). GIP has neuroprotective effects in Alzheimer and Parkinson's disease models. Peptides.

[38] Hoyer D., Bartfai T. (2012). Neuropeptides and neuropeptide receptors: drug targets, and peptide and non-peptide ligands: a tribute to Prof. Dieter Seebach. Chem. Biodivers..

[39] Yao Z., van Velthoven C.T.J., Nguyen T.N., Goldy J., Sedeno-Cortes A.E., Baftizadeh F., Bertagnolli D., Casper T., Chiang M., Crichton K. (2021). A taxonomy of transcriptomic cell types across the isocortex and hippocampal formation. Cell.

[40] Taylor S.R., Santpere G., Weinreb A., Barrett A., Reilly M.B., Xu C., Varol E., Oikonomou P., Glenwinkel L., McWhirter R. (2021). Molecular topography of an entire nervous system. Cell.

[41] Li H., Janssens J., De Waegeneer M., Kolluru S.S., Davie K., Gardeux V., Saelens W., David F.P.A., Brbić M., Spanier K. (2022). Fly Cell Atlas: A single-nucleus transcriptomic atlas of the adult fruit fly. Science.

[42] Tasic B., Yao Z., Graybuck L.T., Smith K.A., Nguyen T.N., Bertagnolli D., Goldy J., Garren E., Economo M.N., Viswanathan S. (2018). Shared and distinct transcriptomic cell types across neocortical areas. Nature.

[43] Smith S.J., Sümbül U., Graybuck L.T., Collman F., Seshamani S., Gala R., Gliko O., Elabbady L., Miller J.A., Bakken T.E. (2019). Single-cell transcriptomic evidence for dense intracortical neuropeptide networks. eLife.

[44] Smith S.J. (2021). Transcriptomic evidence for dense peptidergic networks within forebrains of four widely divergent tetrapods. Curr. Opin. Neurobiol..

[45] Bentley B., Branicky R., Barnes C.L., Chew Y.L., Yemini E., Bullmore E.T., Vértes P.E., Schafer W.R. (2016). The multilayer connectome of Caenorhabditis elegans. PLoS Comput. Biol..

[46] Cook S.J., Jarrell T.A., Brittin C.A., Wang Y., Bloniarz A.E., Yakovlev M.A., Nguyen K.C.Q., Tang L.T., Bayer E.A., Duerr J.S. (2019). Whole-animal connectomes of both Caenorhabditis elegans sexes. Nature.

[47] Varshney L.R., Chen B.L., Paniagua E., Hall D.H., Chklovskii D.B. (2011). Structural properties of the Caenorhabditis elegans neuronal network. PLoS Comput. Biol..

[48] White J.G., Southgate E., Thomson J.N., Brenner S. (1976). The structure of the ventral nerve cord of Caenorhabditis elegans. Philos. Trans. R. Soc. Lond. B Biol. Sci..

[49] White J.G., Southgate E., Thomson J.N., Brenner S. (1986). The structure of the nervous system of the nematode Caenorhabditis elegans. Philos. Trans. R. Soc. Lond. B Biol. Sci..

[50] Watts D.J., Strogatz S.H. (1998). Collective dynamics of 'small-world' networks. Nature.

[51] Bassett D.S., Bullmore E. (2006). Small-world brain networks. Neuroscientist.

[52] Reigl M., Alon U., Chklovskii D.B. (2004). Search for computational modules in the C. elegans brain. BMC Biol..

[53] Alec H., Ann S.B., Danielle S.B. (2021). The growing topology of the <em>C. elegans</em> connectome. bioRxiv.

[54] Pathak A., Chatterjee N., Sinha S. (2020). Developmental trajectory of Caenorhabditis elegans nervous system governs its structural organization. PLoS Comput. Biol..

[55] Pavlovic D.M., Vértes P.E., Bullmore E.T., Schafer W.R., Nichols T.E. (2014). Stochastic blockmodeling of the modules and core of the Caenorhabditis elegans connectome. PLoS One.

[56] Towlson E.K., Vértes P.E., Ahnert S.E., Schafer W.R., Bullmore E.T. (2013). The rich club of the C. elegans neuronal connectome. J. Neurosci..

[57] Schroeter M.S., Charlesworth P., Kitzbichler M.G., Paulsen O., Bullmore E.T. (2015). Emergence of rich-club topology and coordinated dynamics in development of hippocampal functional networks in vitro. J. Neurosci..

[58] van den Heuvel M.P., Sporns O. (2011). Rich-club organization of the human connectome. J. Neurosci..

[59] Milo R., Shen-Orr S., Itzkovitz S., Kashtan N., Chklovskii D., Alon U. (2002). Network motifs: simple building blocks of complex networks. Science.

[60] Alon U. (2007). Network motifs: theory and experimental approaches. Nat. Rev. Genet..

[61] Jordan K.M., Raphael N.-T., Felicia D., Elizabeth P.R., William G.-R. (2021). Circuit motifs and graph properties of connectome development in C. elegans. bioRxiv.

[62] Van Bael S., Zels S., Boonen K., Beets I., Schoofs L., Temmerman L. (2018). A Caenorhabditis elegans Mass spectrometric resource for neuropeptidomics. J. Am. Soc. Mass Spectrom..

[63] McKay F.M., McCoy C.J., Crooks B., Marks N.J., Maule A.G., Atkinson L.E., Mousley A. (2022). In silico analyses of neuropeptide-like protein (NLP) profiles in parasitic nematodes. Int. J. Parasitol..

[64] Hobert O. (2013). The neuronal genome of Caenorhabditis elegans. WormBook.

[65] Foster S.R., Hauser A.S., Vedel L., Strachan R.T., Huang X.P., Gavin A.C., Shah S.D., Nayak A.P., Haugaard-Kedström L.M., Penn R.B. (2019). Discovery of human signaling systems: pairing peptides to G protein-coupled receptors. Cell.

[66] Beets I., Zels S., Vandewyer E., Demeulemeester J., Caers J., Baytemur E., Courtney A., Golinelli L., Hasakioğulları İ., Schafer W.R. (2023). System-wide mapping of peptide-GPCR interactions in C. elegans. Cell Rep..

[67] Chew Y.L., Grundy L.J., Brown A.E.X., Beets I., Schafer W.R. (2018). Neuropeptides encoded by nlp-49 modulate locomotion, arousal and egg-laying behaviours in Caenorhabditis elegans via the receptor SEB-3. Philos. Trans. R. Soc. Lond. B Biol. Sci..

[68] Ghosh D.D., Sanders T., Hong S., McCurdy L.Y., Chase D.L., Cohen N., Koelle M.R., Nitabach M.N. (2016). Neural architecture of hunger-dependent multisensory decision making in C. elegans. Neuron.

[69] Yemini E., Lin A., Nejatbakhsh A., Varol E., Sun R., Mena G.E., Samuel A.D.T., Paninski L., Venkatachalam V., Hobert O. (2021). NeuroPAL: A multicolor atlas for whole-brain neuronal identification in C. elegans. Cell.

[70] Albertson D.G., Thomson J.N. (1976). The pharynx of Caenorhabditis elegans. Philos. Trans. R. Soc. Lond. B Biol. Sci..

[71] Witvliet D., Mulcahy B., Mitchell J.K., Meirovitch Y., Berger D.R., Wu Y., Liu Y., Koh W.X., Parvathala R., Holmyard D. (2021). Connectomes across development reveal principles of brain maturation. Nature.

[72] Brittin C.A., Cook S.J., Hall D.H., Emmons S.W., Cohen N. (2021). A multi-scale brain map derived from whole-brain volumetric reconstructions. Nature.

[73] Moyle M.W., Barnes K.M., Kuchroo M., Gonopolskiy A., Duncan L.H., SenGupta T., Shao L., Guo M., Santella A., Christensen R. (2021). Structural and developmental principles of neuropil assembly in C. elegans. Nature.

[74] Randi F., Sharma A.K., Dvali S., Leifer A.M. (2022). A functional connectivity atlas of $\textit {C. elegans} $ measured by neural activation. Preprint at arXiv.

[75] Pocock R., Hobert O. (2010). Hypoxia activates a latent circuit for processing gustatory information in C. elegans. Nat. Neurosci..

[76] Van Sinay E., Mirabeau O., Depuydt G., Van Hiel M.B., Peymen K., Watteyne J., Zels S., Schoofs L., Beets I. (2017). Evolutionarily conserved TRH neuropeptide pathway regulates growth in Caenorhabditis elegans. Proc. Natl. Acad. Sci. USA.

[77] Bhardwaj A., Thapliyal S., Dahiya Y., Babu K. (2018). FLP-18 functions through the G-protein-coupled receptors NPR-1 and NPR-4 to modulate reversal length in Caenorhabditis elegans. J. Neurosci..

[78] Lindemans M., Liu F., Janssen T., Husson S.J., Mertens I., Gäde G., Schoofs L. (2009). Adipokinetic hormone signaling through the gonadotropin-releasing hormone receptor modulates egg-laying in Caenorhabditis elegans. Proc. Natl. Acad. Sci. USA.

[79] Cook S.J., Crouse C.M., Yemini E., Hall D.H., Emmons S.W., Hobert O. (2020). The connectome of the Caenorhabditis elegans pharynx. J. Comp. Neurol..

[80] Vidal B., Gulez B., Cao W.X., Leyva-Díaz E., Reilly M.B., Tekieli T., Hobert O. (2022). The enteric nervous system of the C. elegans pharynx is specified by the Sine oculis-like homeobox gene ceh-34. eLife.

[81] Kaplan H.S., Nichols A.L.A., Zimmer M. (2018). Sensorimotor integration in Caenorhabditis elegans: a reappraisal towards dynamic and distributed computations. Philos. Trans. R. Soc. Lond. B Biol. Sci..

[82] Zhou S., Mondragon R.J. (2004). The rich-club phenomenon in the Internet topology. IEEE Commun. Lett..

[83] Pereira L., Kratsios P., Serrano-Saiz E., Sheftel H., Mayo A.E., Hall D.H., White J.G., LeBoeuf B., Garcia L.R., Alon U. (2015). A cellular and regulatory map of the cholinergic nervous system of C. elegans. eLife.

[84] Niu L., Li Y., Zong P., Liu P., Shui Y., Chen B., Wang Z.W. (2020). Melatonin promotes sleep by activating the BK channel in C. elegans. Proc. Natl. Acad. Sci. USA.

[85] Hums I., Riedl J., Mende F., Kato S., Kaplan H.S., Latham R., Sonntag M., Traunmüller L., Zimmer M. (2016). Regulation of two motor patterns enables the gradual adjustment of locomotion strategy in Caenorhabditis elegans. eLife.

[86] Marquina-Solis J., Vandewyer E., Hawk J., Colón-Ramos D.A., Beets I., Bargmann C.I. (2022). Peptidergic signaling controls the dynamics of sickness behavior in Caenorhabditis elegans. bioRxiv.

[87] Betzel R.F., Medaglia J.D., Bassett D.S. (2018). Diversity of meso-scale architecture in human and non-human connectomes. Nat. Commun..

[88] Wen Q., Po M.D., Hulme E., Chen S., Liu X., Kwok S.W., Gershow M., Leifer A.M., Butler V., Fang-Yen C. (2012). Proprioceptive coupling within motor neurons drives C. elegans forward locomotion. Neuron.

[89] Schafer W.R. (2015). Mechanosensory molecules and circuits in C. elegans. Pflugers Arch..

[90] Fenk L.A., de Bono M. (2017). Memory of recent oxygen experience switches pheromone valence in Caenorhabditis elegans. Proc. Natl. Acad. Sci. USA.

[91] Laurent P., Soltesz Z., Nelson G.M., Chen C., Arellano-Carbajal F., Levy E., de Bono M. (2015). Decoding a neural circuit controlling global animal state in C. elegans. eLife.

[92] Zimmer M., Gray J.M., Pokala N., Chang A.J., Karow D.S., Marletta M.A., Hudson M.L., Morton D.B., Chronis N., Bargmann C.I. (2009). Neurons detect increases and decreases in oxygen levels using distinct guanylate cyclases. Neuron.

[93] Busch K.E., Laurent P., Soltesz Z., Murphy R.J., Faivre O., Hedwig B., Thomas M., Smith H.L., de Bono M. (2012). Tonic signaling from O(2) sensors sets neural circuit activity and behavioral state. Nat. Neurosci..

[94] Haspel G., O'Donovan M.J., Hart A.C. (2010). Motoneurons dedicated to either forward or backward locomotion in the nematode Caenorhabditis elegans. J. Neurosci..

[95] Tolstenkov O., Van der Auwera P., Steuer Costa W., Bazhanova O., Gemeinhardt T.M., Bergs A.C., Gottschalk A. (2018). Functionally asymmetric motor neurons contribute to coordinating locomotion of Caenorhabditis elegans. eLife.

[96] Butler V.J., Branicky R., Yemini E., Liewald J.F., Gottschalk A., Kerr R.A., Chklovskii D.B., Schafer W.R. (2015). A consistent muscle activation strategy underlies crawling and swimming in Caenorhabditis elegans. J. R. Soc. Interface.

[97] Faumont S., Rondeau G., Thiele T.R., Lawton K.J., McCormick K.E., Sottile M., Griesbeck O., Heckscher E.S., Roberts W.M., Doe C.Q. (2011). An image-free opto-mechanical system for creating virtual environments and imaging neuronal activity in freely moving Caenorhabditis elegans. PLoS One.

[98] Gao S., Guan S.A., Fouad A.D., Meng J., Kawano T., Huang Y.C., Li Y., Alcaire S., Hung W., Lu Y. (2018). Excitatory motor neurons are local oscillators for backward locomotion. eLife.

[99] Kato S., Kaplan H.S., Schrödel T., Skora S., Lindsay T.H., Yemini E., Lockery S., Zimmer M. (2015). Global brain dynamics embed the motor command sequence of Caenorhabditis elegans. Cell.

[100] Worrell J.C., Rumschlag J., Betzel R.F., Sporns O., Mišić B. (2018). Optimized connectome architecture for sensory-motor integration. Netw. Neurosci..

[101] Williams E.A., Verasztó C., Jasek S., Conzelmann M., Shahidi R., Bauknecht P., Mirabeau O., Jékely G. (2017). Synaptic and peptidergic connectome of a neurosecretory center in the annelid brain. eLife.

[102] Rabinowitch I., Laurent P., Zhao B., Walker D., Beets I., Schoofs L., Bai J., Schafer W.R., Treinin M. (2016). Neuropeptide-Driven Cross-Modal Plasticity following Sensory Loss in Caenorhabditis elegans. PLoS Biol..

[103] Gordus A., Pokala N., Levy S., Flavell S.W., Bargmann C.I. (2015). Feedback from network states generates variability in a probabilistic olfactory circuit. Cell.

[104] Turek M., Besseling J., Spies J.P., König S., Bringmann H. (2016). Sleep-active neuron specification and sleep induction require FLP-11 neuropeptides to systemically induce sleep. eLife.

[105] Xu Y., Zhang L., Liu Y., Topalidou I., Hassinan C., Ailion M., Zhao Z., Wang T., Chen Z., Bai J. (2021). Dopamine receptor DOP-1 engages a sleep pathway to modulate swimming in C. elegans. iScience.

[106] Michael F.P., Sara N.F., Deanna B., Vasin D., Yevgenia K. (2021). Peptidergic modulation of fear responses by the Edinger-Westphal nucleus. bioRxiv.

[107] Kim C.K., Cho K.F., Kim M.W., Ting A.Y. (2019). Luciferase-LOV BRET enables versatile and specific transcriptional readout of cellular protein-protein interactions. eLife.

[108] Kim M.W., Wang W., Sanchez M.I., Coukos R., von Zastrow M., Ting A.Y. (2017). Time-gated detection of protein-protein interactions with transcriptional readout. eLife.

[109] Chen R., Wu X., Jiang L., Zhang Y. (2017). Single-cell RNA-seq reveals hypothalamic cell diversity. Cell Rep..

[110] Terauchi A., Johnson-Venkatesh E.M., Bullock B., Lehtinen M.K., Umemori H. (2016). Retrograde fibroblast growth factor 22 (FGF22) signaling regulates insulin-like growth factor 2 (IGF2) expression for activity-dependent synapse stabilization in the mammalian brain. eLife.

[111] Root C.M., Ko K.I., Jafari A., Wang J.W. (2011). Presynaptic facilitation by neuropeptide signaling mediates odor-driven food search. Cell.

[112] Horowitz L.B., Brandt J.P., Ringstad N. (2019). Repression of an activity-dependent autocrine insulin signal is required for sensory neuron development in C. elegans. Development.

[113] Mardinly A.R., Spiegel I., Patrizi A., Centofante E., Bazinet J.E., Tzeng C.P., Mandel-Brehm C., Harmin D.A., Adesnik H., Fagiolini M. (2016). Sensory experience regulates cortical inhibition by inducing IGF1 in VIP neurons. Nature.

[114] Choi C., Cao G., Tanenhaus A.K., McCarthy E.V., Jung M., Schleyer W., Shang Y., Rosbash M., Yin J.C., Nitabach M.N. (2012). Autoreceptor control of peptide/neurotransmitter corelease from PDF neurons determines allocation of circadian activity in drosophila. Cell Rep..

[115] Yoshioka J., Prince R.N., Huang H., Perkins S.B., Cruz F.U., MacGillivray C., Lauffenburger D.A., Lee R.T. (2005). Cardiomyocyte hypertrophy and degradation of connexin43 through spatially restricted autocrine/paracrine heparin-binding EGF. Proc. Natl. Acad. Sci. USA.

[116] Tse L.H., Wong Y.H. (2019). GPCRs in autocrine and paracrine regulations. Front. Endocrinol. (Lausanne).

[117] Yemini E., Jucikas T., Grundy L.J., Brown A.E., Schafer W.R. (2013). A database of Caenorhabditis elegans behavioral phenotypes. Nat. Methods.

[118] Brown A.E., Yemini E.I., Grundy L.J., Jucikas T., Schafer W.R. (2013). A dictionary of behavioral motifs reveals clusters of genes affecting Caenorhabditis elegans locomotion. Proc. Natl. Acad. Sci. USA.

[119] Avery L., Horvitz H.R. (1989). Pharyngeal pumping continues after laser killing of the pharyngeal nervous system of C. elegans. Neuron.

[120] Forrester W.C., Garriga G. (1997). Genes necessary for C. elegans cell and growth cone migrations. Development.

[121] Winding M., Pedigo B.D., Barnes C.L., Patsolic H.G., Park Y., Kazimiers T., Fushiki A., Andrade I.V., Khandelwal A., Valdes-Aleman J. (2023). The connectome of an insect brain. Science.

[122] Brenner S. (1974). The genetics of *Caenorhabditis elegans*. Genetics.

[123] Sun H., Hobert O. (2021). Temporal transitions in the post-mitotic nervous system of Caenorhabditis elegans. Nature.

[124] Tekieli T., Yemini E., Nejatbakhsh A., Wang C., Varol E., Fernandez R.W., Masoudi N., Paninski L., Hobert O. (2021). Visualizing the organization and differentiation of the male-specific nervous system of C. elegans. Development.

[125] Reilly M.B., Tekieli T., Cros C., Aguilar G.R., Lao J., Toker I.A., Vidal B., Leyva-Díaz E., Bhattacharya A., Cook S.J. (2022). Widespread employment of conserved C. elegans homeobox genes in neuronal identity specification. PLOS Genet..

[126] Cros C., Hobert O. (2022). Caenorhabditis elegans sine oculis/SIX-type homeobox genes act as homeotic switches to define neuronal subtype identities. Proc. Natl. Acad. Sci. USA.

[127] Rubinov M., Sporns O. (2010). Complex network measures of brain connectivity: uses and interpretations. Neuroimage.

[128] Shannon P., Markiel A., Ozier O., Baliga N.S., Wang J.T., Ramage D., Amin N., Schwikowski B., Ideker T. (2003). Cytoscape: a software environment for integrated models of biomolecular interaction networks. Genome Res..

[129] De Domenico M., Porter M.A., Arenas A. (2015). MuxViz: a tool for multilayer analysis and visualization of networks. J. Complex Netw..

[130] Altun Z.F., Herndon L.A., Wolkow C.A., Crocker C., Lints R., Hall D.H. (2002).

[131] Beets I., Zels S., Vandewyer E., Demeulemeester J., Caers J., Baytemur E., Schafer W.R., Vértes P.E., Mirabeau O., Schoofs L. (2022). System-wide mapping of neuropeptide-GPCR interactions in C. elegans. bioRxiv.

[132] Fisher R.A. (1934).

[133] Storey J.D. (2002). A direct approach to false discovery rates. J. R. Stat. Soc. B.

[134] Bounova G., de Weck O. (2012). Overview of metrics and their correlation patterns for multiple-metric topology analysis on heterogeneous graph ensembles. Phys. Rev. E Stat. Nonlin. Soft Matter Phys..

