# Supplementary material for: The neuropeptidergic connectome of C. elegans
Source: Neuron. Author manuscript; Available in PMC 2024 Jan 4. (PMC7615469; doi:10.1016/j.neuron.2023.09.043)
Supplement: Document S1. Figures S1–S12. [file EMS192830-supplement-Document_S1__Figures_S1_S12_.pdf]

Neuron, Volume *111*

Supplemental information

The neuropeptidergic connectome of *C. elegans*

Lidia Ripoll-Sánchez, Jan Watteyne, HaoSheng Sun, Robert Fernandez, Seth R. Taylor, Alexis Weinreb, Barry L. Bentley, Marc Hammarlund, David M. Miller III, Oliver Hobert, Isabel Beets, Petra E. Vértes, and William R. Schafer

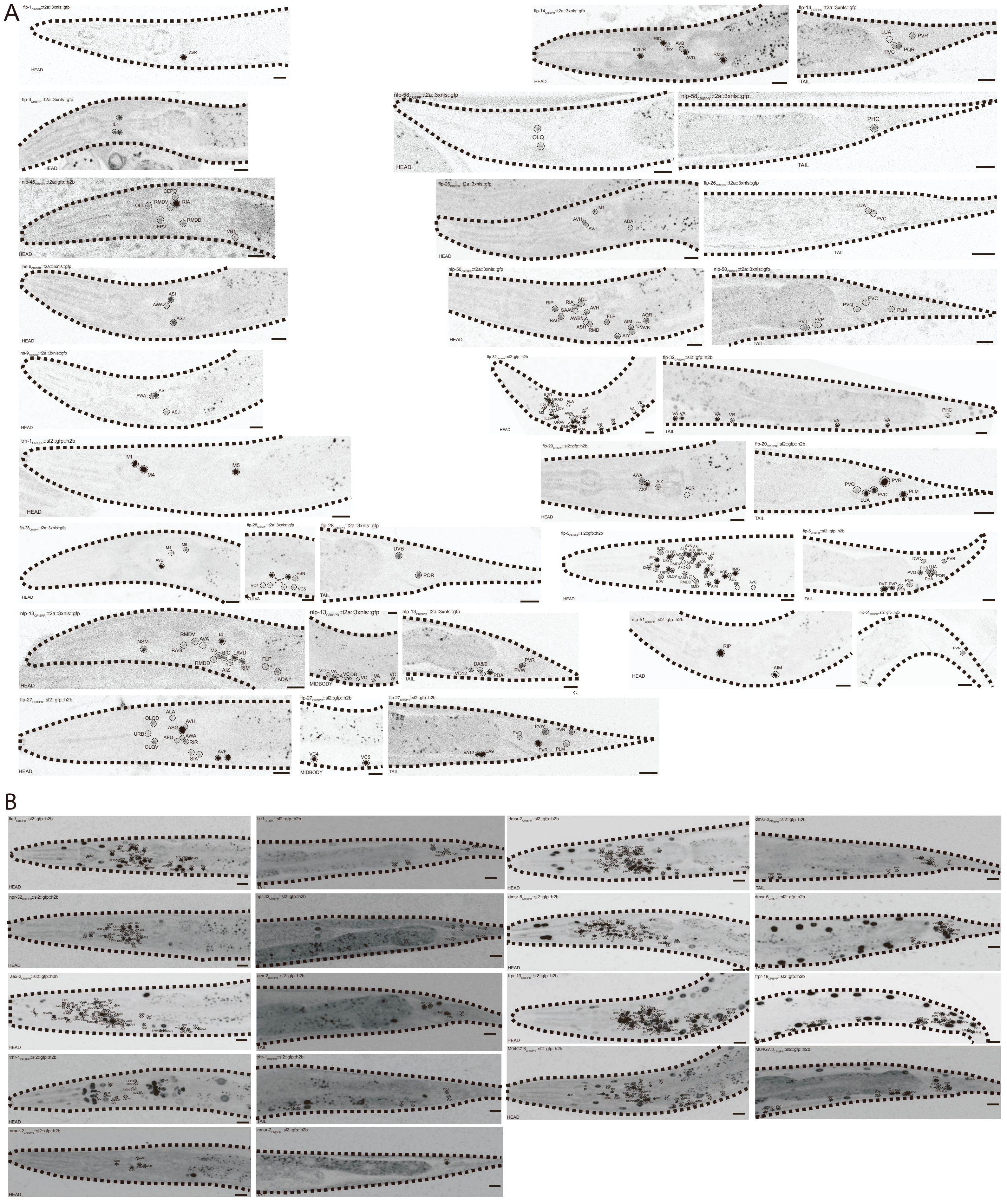
A
B
HEAD
TAIL
MIDBODY
VULVA

Figure S1. Single-copy GFP genomic knock-in reporters for the expression of representative neuropeptide and GPCR genes. Related to Figure 2. GFP-positive neurons were identified using the NeuroPAL multicolor transgene (Yemini et al., 2021). Figure pictures segments (head, tail, vulva and midbody) showing neuronal expression, within which individual neurons are circled and labeled. The names of the reporter-expressing genes are indicated on top of each figure and the names of the segment at the bottom of the figure. Scale bars represent 10 μm.

(A) Fluorescent GFP reporters shown for the expression of 17 neuropeptide precursor genes.
(B) Fluorescent GFP reporters shown shown for the expression of 9 GPCR precursor genes.

A

Identities of rows in Figure 3			Amount of neurons that require each type of connection								
			GPCR expression (panel C)				cognate NPP expression (panel D)				
Row number	GPCR (panel C)	Cognate NPP (panel D)	Mid range	No contact (inside bundle)	Contact (inside bundle)	Contact (inside NR)	Mid range	No contact (inside bundle)	Contact (inside bundle)	Contact (inside NR)	Contact (inside NR)
1	TRHR-1	NLP-54	2	5	34	6	0	0	7	2	2
2	SPRR-2	NLP-38	0	0	4	11	0	13	32	5	5
3	SPRR-1	NLP-42	5	8	1	27	0	0	0	5	5
4	SEB-3	NLP-49	0	4	65	8	1	4	0	15	15
5	PDFR-1	NLP-37	4	25	54	73	0	1	0	14	14
6	NPR-8	FLP-27	0	0	8	30	1	6	7	23	23
7	NPR-42	NLP-3	0	1	2	9	8	3	1	28	28
8	NPR-35	NLP-10	0	2	6	14	9	12	6	27	27
9	NPR-34	SNET-1	3	1	63	7	0	10	0	14	14
10	NPR-32	NLP-64	2	16	3	28	0	3	2	17	17
11	NPR-3	FLP-15	2	8	10	23	0	3	26	17	17
12	NPR-22	NLP-72	0	6	6	0	0	2	7	0	0
13	NPR-11	FLP-34	0	2	3	13	9	8	2	19	19
14	GNRR-3	NLP-23	0	10	0	1	0	0	0	2	2
15	GNRR-1	NLP-47	0	1	32	7	0	29	0	31	31
16	FRPR-9	FLP-19	0	9	2	27	2	3	3	27	27
17	FRPR-6	FLP-33	0	19	0	1	0	0	2	2	2
18	FRPR-3	FLP-20	0	6	5	8	0	5	6	8	8
19	FRPR-18	FLP-2	0	3	6	24	0	5	10	21	21
20	FRPR-15	FLP-8	8	42	2	1	0	2	1	2	2
21	DMSR-8	FLP-12	0	10	21	16	0	3	7	12	12
22	DMSR-3	FLP-14	5	0	1	14	0	4	6	20	20
23	AEX-2	NLP-40	5	0	1	12	0	2	1	13	13

B

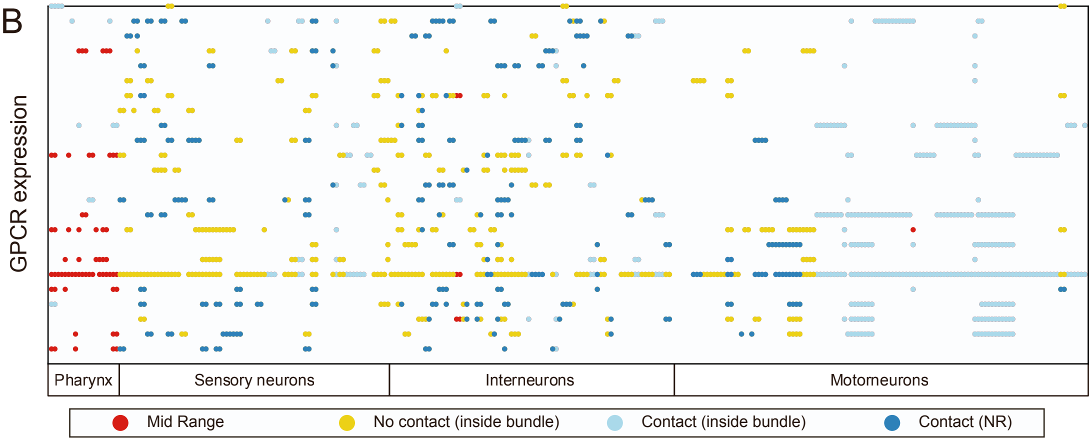

C

Identities of rows in Figure S2B											
Row number	GPCR	Row number	GPCR	Row number	GPCR	Row number	GPCR	Row number	GPCR	Row number	GPCR
1	TRHR-1	8	NPR-8	15	NPR-35	22	NPR-10	29	FRPR-7	36	DMSR-6
2	TKR-2	9	NPR-5	16	NPR-34	23	NPR-1	30	FRPR-6	37	DMSR-5
3	TKR-1	10	NPR-43	17	NPR-32	24	NMUR-2	31	FRPR-3	38	DMSR-3
4	SPRR-2	11	NPR-42	18	NPR-3	25	NMUR-1	32	FRPR-18	39	DMSR-2
5	SPRR-1	12	NPR-41	19	NPR-22	26	GNRR-3	33	FRPR-16	40	CKR-2
6	SEB-3	13	NPR-4	20	NPR-2	27	GNRR-1	34	FRPR-15	41	CKR-1
7	PDFR-1	14	NPR-37	21	NPR-11	28	FRPR-9	35	DMSR-8	42	AEX-2

Figure S2. Analysis of neuropeptide and receptor expression allows assessment of the spatial scale of neuropeptide signaling. Related to Figure 3

(A) Gene identities of the GPCRs (Figure 3C) and neuropeptides (Figure 3D) represented in the rows of the expression matrices of specific NPP-GPCR interactions *in vitro* (Figure 3C, D*)*. Columns indicate the number of neurons that require each range of diffusion distance to allow neuropeptidergic communication for that NPP-GPCR pair. Table also shown in Figure S2A ID table.

(B) Complete expression matrix for the 42 GPCRs activated *in vitro* by a single neuropeptide ligand. Out of this subset 19 GPCRs bind non-specific neuropeptides that also bind other GPCRs.

(C) Gene identities of the GPCRs included in the expression matrix of Figure S2B. Table also shown in Figure S2B ID table.

Short-range NPP-GPCR networks EC_{50} 500nM

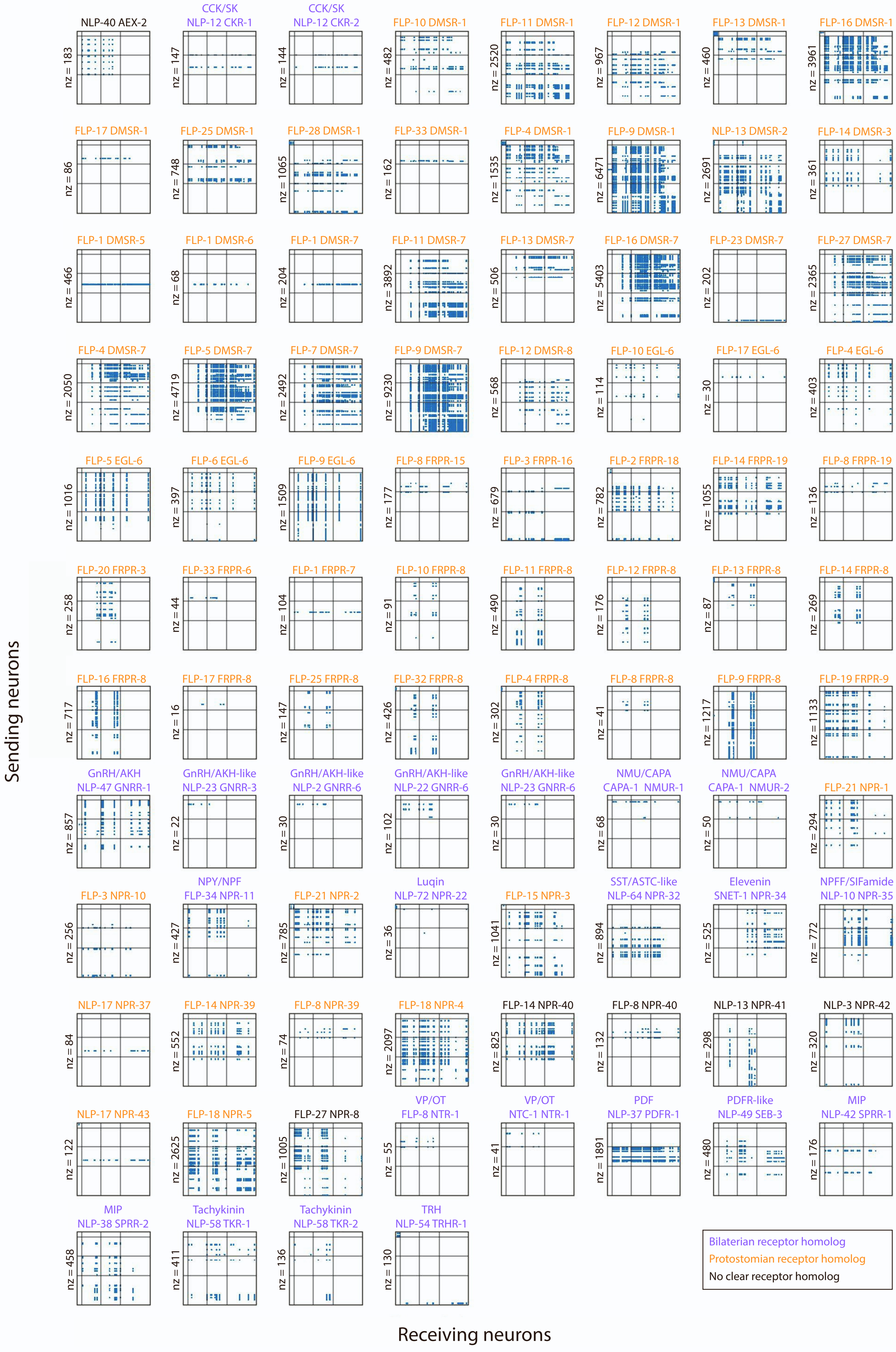

Figure S3. Adjacency matrix representation of the 92 individual NPP-GPCR pair networks considering short-range diffusion connections. Related to Figure 4 and 5. Adjacency matrices rows and columns are sorted by neuron type like Figure 5, line divisions for pharyngeal neurons, sensory neurons, interneurons and motorneurons. The number of connections (nz) is shown on the lateral side of each matrix. Evolutionary relationships for the different NPP-GPCR systems are color-coded as classified by Mirabeau and Joly (2013), Elphick et al. (2018), and Beets et al. (2023): NPP-GPCR systems that are ancestral to bilaterian animals are indicated in purple, while those ancestral to protostomian are orange. Nematode-specific NPP-GPCRs without clear receptor homologs are colored black. Short forms mean: cholecystokinin / sulfakinin (CCK/SK), gonadotropin-releasing hormone / adipokinetic hormone (GnRH/AKH), neuromedin U /capability (NMU/CAPA), neuropeptide Y / neuropeptide F (NPY/NPF), Somatostatin / allatostatin-C (SST/ASTC), neuropeptide FF / SIFamide (NPFF/SIFa), vasopressin / oxytocin (VP/OT), pigment dispersing factor (PDF), pigment dispersing factor receptor-like (PDFR-like), myoinhibiting peptide (MIP), thyrotropin-releasing hormone (TRH).

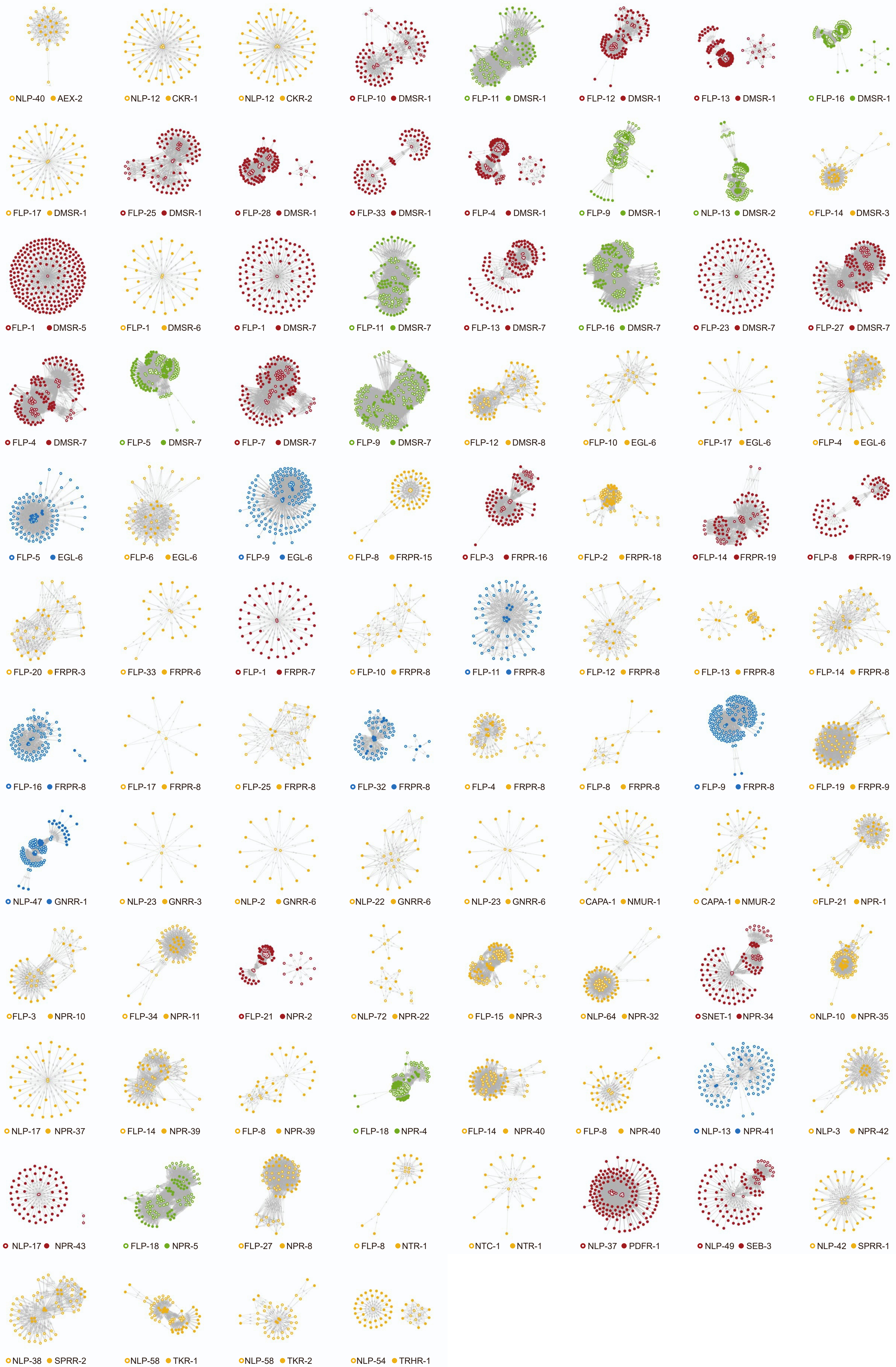
NLP-40 AEX-2
NLP-12 CKR-1
NLP-12 CKR-2
FLP-10 DMSR-1
FLP-11 DMSR-1
FLP-12 DMSR-1
FLP-13 DMSR-1
FLP-16 DMSR-1
FLP-17 DMSR-1
FLP-25 DMSR-1
FLP-28 DMSR-1
FLP-33 DMSR-1
FLP-4 DMSR-1
FLP-9 DMSR-1
NLP-13 DMSR-2
FLP-14 DMSR-3
FLP-1 DMSR-5
FLP-1 DMSR-6
FLP-1 DMSR-7
FLP-11 DMSR-7
FLP-13 DMSR-7
FLP-16 DMSR-7
FLP-23 DMSR-7
FLP-27 DMSR-7
FLP-4 DMSR-7
FLP-5 DMSR-7
FLP-7 DMSR-7
FLP-9 DMSR-7
FLP-12 DMSR-8
FLP-10 EGL-6
FLP-17 EGL-6
FLP-4 EGL-6
FLP-5 EGL-6
FLP-6 EGL-6
FLP-9 EGL-6
FLP-8 FRPR-15
FLP-3 FRPR-16
FLP-2 FRPR-18
FLP-14 FRPR-19
FLP-8 FRPR-19
FLP-20 FRPR-3
FLP-33 FRPR-6
FLP-1 FRPR-7
FLP-10 FRPR-8
FLP-11 FRPR-8
FLP-12 FRPR-8
FLP-13 FRPR-8
FLP-14 FRPR-8
FLP-16 FRPR-8
FLP-17 FRPR-8
FLP-25 FRPR-8
FLP-32 FRPR-8
FLP-4 FRPR-8
FLP-8 FRPR-8
FLP-9 FRPR-8
FLP-19 FRPR-9
NLP-47 GNRR-1
NLP-23 GNRR-3
NLP-2 GNRR-6
NLP-22 GNRR-6
NLP-23 GNRR-6
CAPA-1 NMUR-1
CAPA-1 NMUR-2
FLP-21 NPR-1
FLP-3 NPR-10
FLP-34 NPR-11
FLP-21 NPR-2
NLP-72 NPR-22
FLP-15 NPR-3
NLP-64 NPR-32
SNET-1 NPR-34
NLP-10 NPR-35
NLP-17 NPR-37
FLP-14 NPR-39
FLP-8 NPR-39
FLP-18 NPR-4
FLP-14 NPR-40
FLP-8 NPR-40
NLP-13 NPR-41
NLP-3 NPR-42
NLP-17 NPR-43
FLP-18 NPR-5
FLP-27 NPR-8
FLP-8 NTR-1
NTC-1 NTR-1
NLP-37 PDFR-1
NLP-49 SEB-3
NLP-42 SPRR-1
NLP-38 SPRR-2
NLP-58 TKR-1
NLP-58 TKR-2
NLP-54 TRHR-1

Figure S4. Graphical representation of the 92 individual NPP-GPCR pair networks considering short-range diffusion connections. Related to Figure 4 and 5. Networks are sorted in the same order as in Figure S3 for comparison. Colors given by assortativity analysis shown in Figure 4; yellow indicates local, green pervasive, blue integrative and red promiscuous networks. Open circles indicate nodes that express the neuropeptide and closed circles nodes that express the receptor.

Mid-range NPP-GPCR networks EC_{50} 500nM

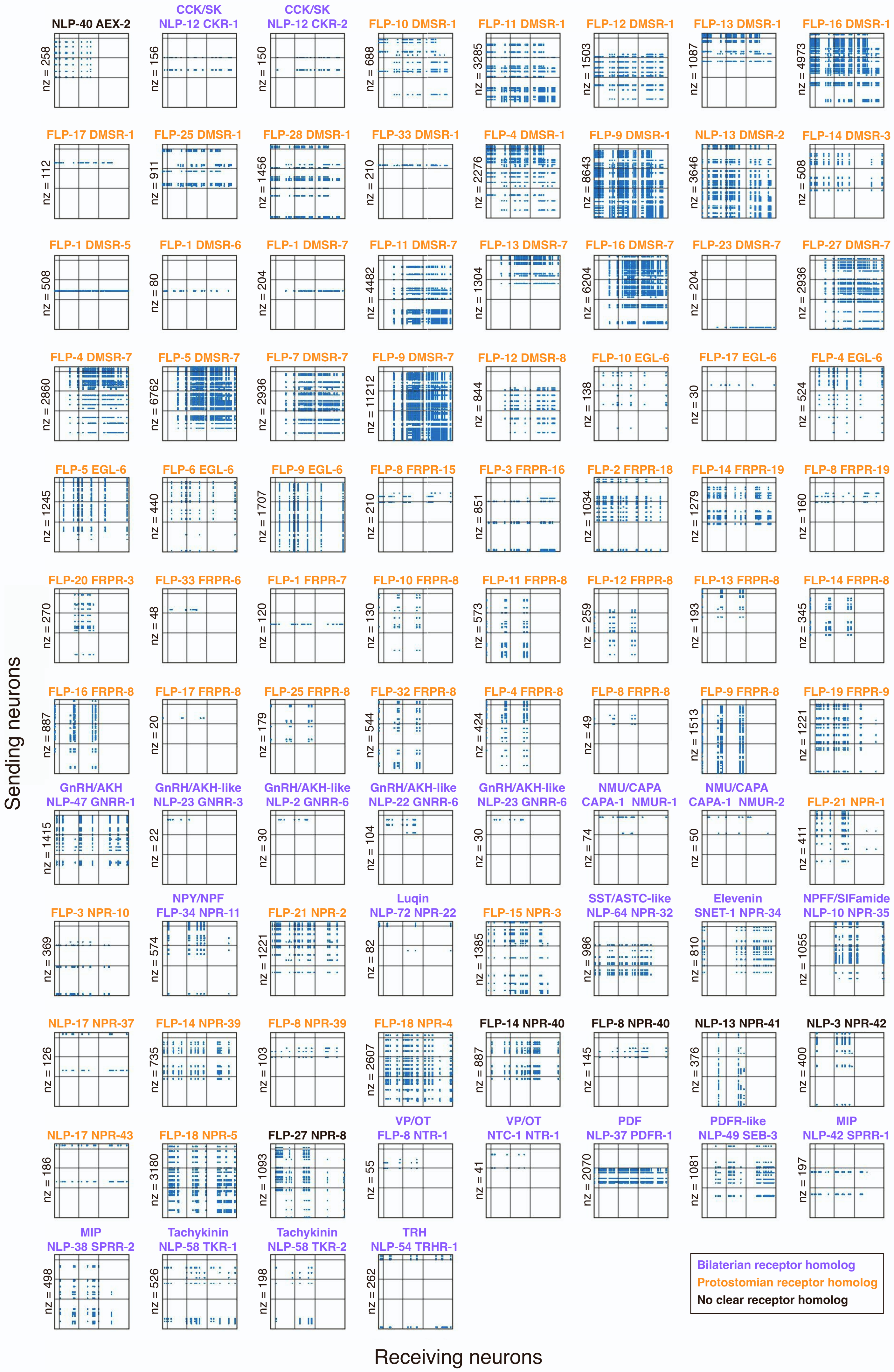

Figure S5. Adjacency matrix representation of the 92 individual NPP-GPCR pair networks considering mid-range diffusion connections. Related to Figure 4 and 5. Adjacency matrices rows and columns are sorted by neuron type like Figure 5, line divisions for pharyngeal neurons, sensory neurons, interneurons and motorneurons. The number of connections (nz) is shown on the lateral side of each matrix. Evolutionary relationships for the different NPP-GPCR systems are color-coded as classified by Mirabeau and Joly (2013), Elphick et al. (2018), and Beets et al. (2023): NPP-GPCR systems that are ancestral to bilaterian animals are indicated in purple, while those ancestral to protostomian are orange. Nematode-specific NPP-GPCRs without clear receptor homologs are colored black. Short forms mean: cholecystokinin / sulfakinin (CCK/SK), gonadotropin-releasing hormone / adipokinetic hormone (GnRH/AKH), neuromedin U /capability (NMU/CAPA), neuropeptide Y / neuropeptide F (NPY/NPF), Somatostatin / allatostatin-C (SST/ASTC), neuropeptide FF / SIFamide (NPFF/SIFa), vasopressin / oxytocin (VP/OT), pigment dispersing factor (PDF), pigment dispersing factor receptor-like (PDFR-like), myoinhibiting peptide (MIP), thyrotropin-releasing hormone (TRH).

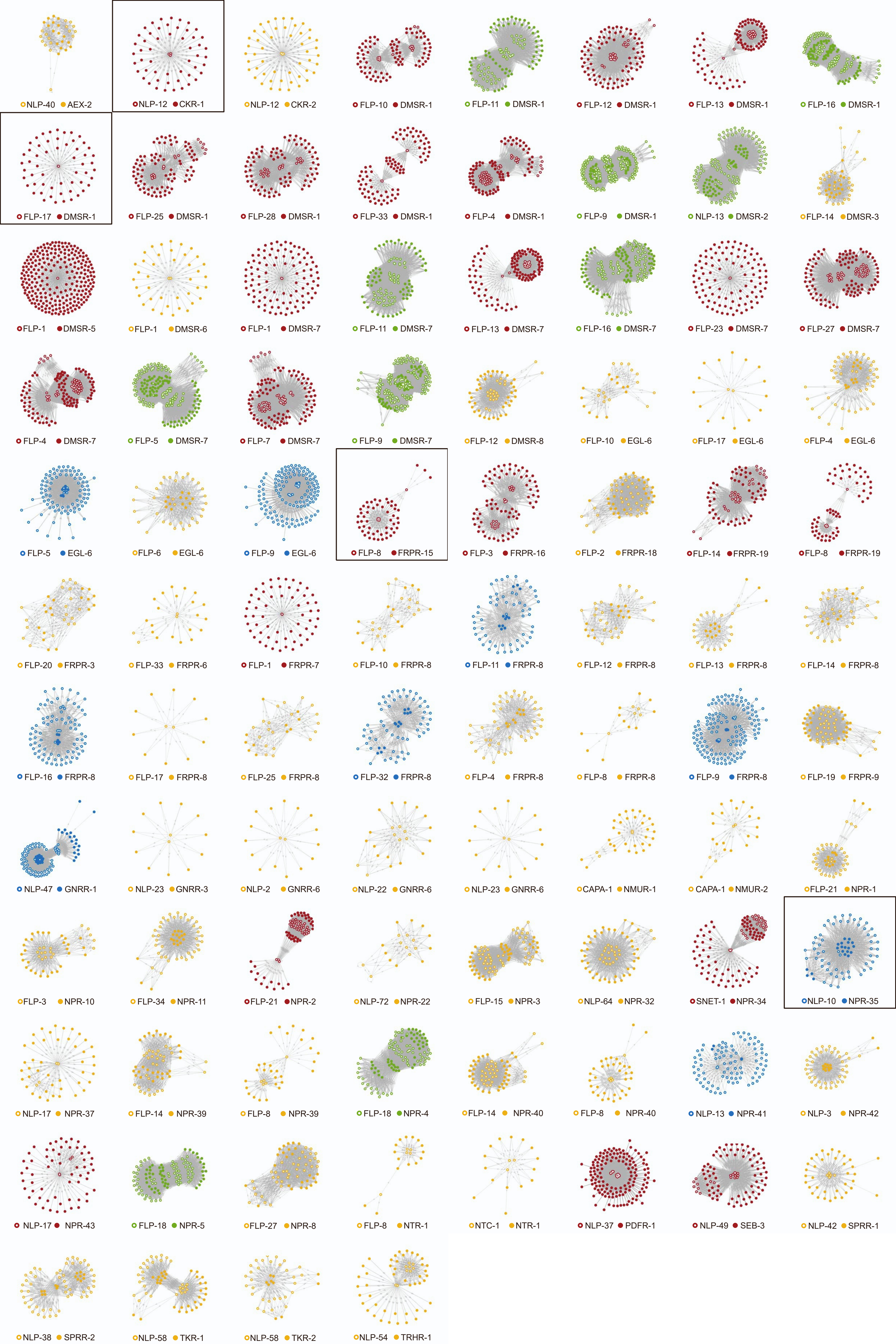
NLP-40 AEX-2
NLP-12 CKR-1
NLP-12 CKR-2
FLP-10 DMSR-1
FLP-11 DMSR-1
FLP-12 DMSR-1
FLP-13 DMSR-1
FLP-16 DMSR-1
FLP-17 DMSR-1
FLP-25 DMSR-1
FLP-28 DMSR-1
FLP-33 DMSR-1
FLP-4 DMSR-1
FLP-9 DMSR-1
NLP-13 DMSR-2
FLP-14 DMSR-3
FLP-1 DMSR-5
FLP-1 DMSR-6
FLP-1 DMSR-7
FLP-11 DMSR-7
FLP-13 DMSR-7
FLP-16 DMSR-7
FLP-23 DMSR-7
FLP-27 DMSR-7
FLP-4 DMSR-7
FLP-5 DMSR-7
FLP-7 DMSR-7
FLP-9 DMSR-7
FLP-12 DMSR-8
FLP-10 EGL-6
FLP-17 EGL-6
FLP-4 EGL-6
FLP-5 EGL-6
FLP-6 EGL-6
FLP-9 EGL-6
FLP-8 FRPR-15
FLP-3 FRPR-16
FLP-2 FRPR-18
FLP-14 FRPR-19
FLP-8 FRPR-19
FLP-20 FRPR-3
FLP-33 FRPR-6
FLP-1 FRPR-7
FLP-10 FRPR-8
FLP-11 FRPR-8
FLP-12 FRPR-8
FLP-13 FRPR-8
FLP-14 FRPR-8
FLP-16 FRPR-8
FLP-17 FRPR-8
FLP-25 FRPR-8
FLP-32 FRPR-8
FLP-4 FRPR-8
FLP-8 FRPR-8
FLP-9 FRPR-8
FLP-19 FRPR-9
NLP-47 GNRR-1
NLP-23 GNRR-3
NLP-2 GNRR-6
NLP-22 GNRR-6
NLP-23 GNRR-6
CAPA-1 NMUR-1
CAPA-1 NMUR-2
FLP-21 NPR-1
FLP-3 NPR-10
FLP-34 NPR-11
FLP-21 NPR-2
NLP-72 NPR-22
FLP-15 NPR-3
NLP-64 NPR-32
SNET-1 NPR-34
NLP-10 NPR-35
NLP-17 NPR-37
FLP-14 NPR-39
FLP-8 NPR-39
FLP-18 NPR-4
FLP-14 NPR-40
FLP-8 NPR-40
NLP-13 NPR-41
NLP-3 NPR-42
NLP-17 NPR-43
FLP-18 NPR-5
FLP-27 NPR-8
FLP-8 NTR-1
NTC-1 NTR-1
NLP-37 PDFR-1
NLP-49 SEB-3
NLP-42 SPRR-1
NLP-38 SPRR-2
NLP-58 TKR-1
NLP-58 TKR-2
NLP-54 TRHR-1

Figure S6. Graphical representation of the 92 individual NPP-GPCR pair networks considering mid-range diffusion connections. Related to Figure 4 and 5. Networks are sorted in the same order as in Figure S3 for comparison. Colors given by assortativity analysis shown in Figure 4; yellow indicates local, green pervasive, blue integrative and red promiscuous networks. Open circles indicate nodes that express the neuropeptide and closed circles nodes that express the receptor. Networks that change assortativity between short and mid-range spatial models of neuropeptide transmission are highlighted by a black square.

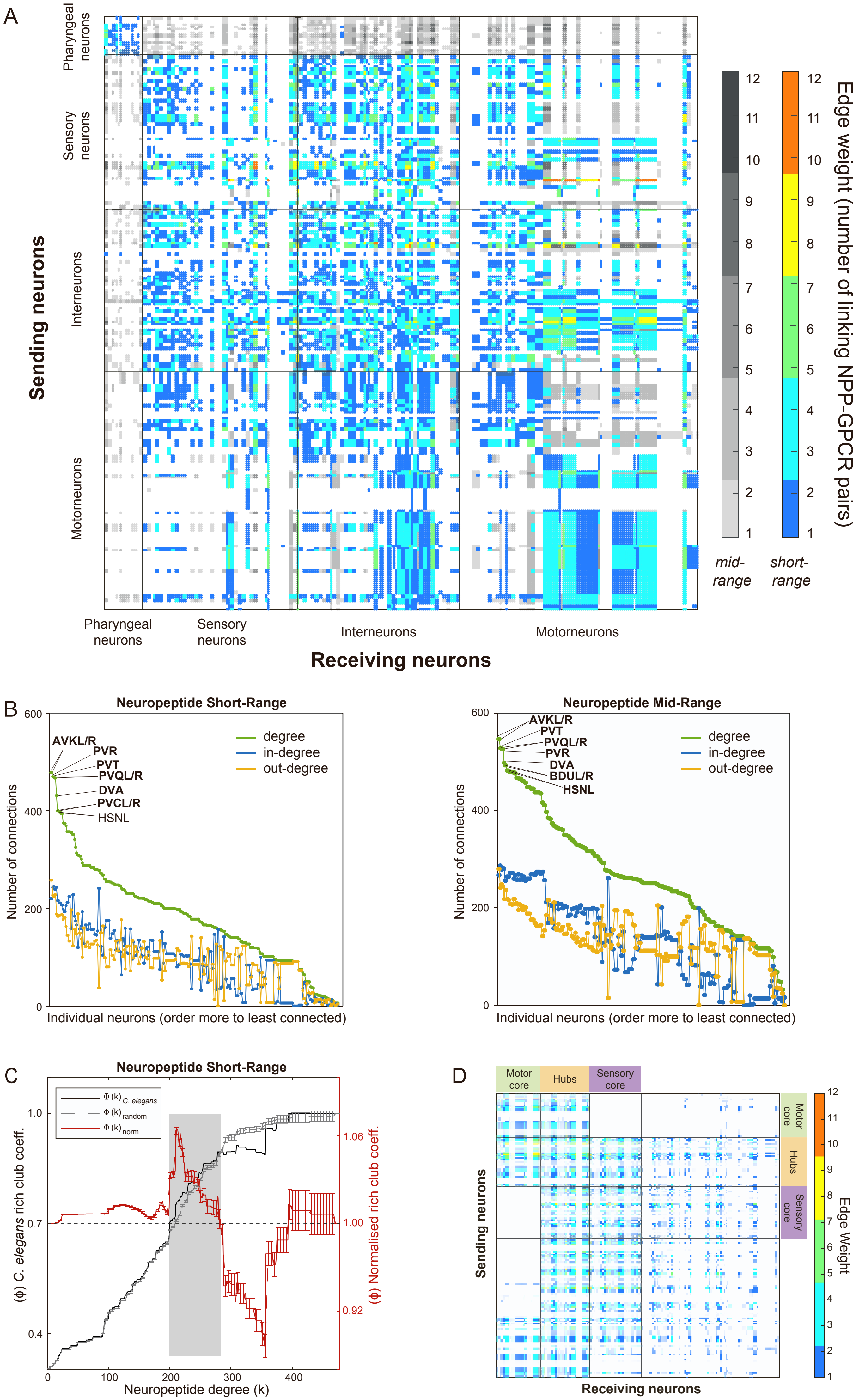
A
Pharyngeal neurons
Sensory neurons
Interneurons
Motorneurons
Sending neurons
Pharyngeal neurons
Sensory neurons
Interneurons
Motorneurons
Receiving neurons
Edge weight (number of linking NPP-GPCR pairs)
mid-range
short-range
B
Neuropeptide Short-Range
Number of connections
AVKL/R
PVR
PVT
PVQL/R
DVA
PVCL/R
HSNL
degree
in-degree
out-degree
Individual neurons (order more to least connected)
Neuropeptide Mid-Range
AVKL/R
PVT
PVQL/R
PVR
DVA
BDUL/R
HSNL
C
Neuropeptide Short-Range
Φ (k) C. elegans
Φ (k) random
Φ (k) norm
(ϕ) C. elegans rich club coeff.
(ϕ) Normalised rich club coeff.
Neuropeptide degree (k)
D
Motor core
Hubs
Sensory core
Sending neurons
Receiving neurons
Edge Weight

Figure S7. Restricting the EC_{50} threshold for biochemical neuropeptide-GPCR interaction to 100 nM retains the overall structure of the neuropeptidergic connectome. Related to Figure 5, 6, and 7.

(A) Adjacency matrix representation of the aggregate 67 NPP-GPCR pair network below 100 nM EC_{50} threshold considering short-range (color) and mid-range (gray) diffusion models. Related to Figure 5 and Table S5.

(B) Degree distribution of the aggregate neuropeptide network below 100 nM EC_{50} threshold considering short-range (left) and mid-range (right) diffusion models. Degree (sum of incoming and outgoing connections) is shown in green, the in-degree (sum of incoming connections) is shown in blue and the out-degree (sum of outgoing connections) is shown in yellow. The 10 hubs with the highest degree are labelled, the hubs that are conserved between this network and the one with 500nM EC_{50} threshold are shown in bold, portraying high conservation. Related to Figure 6B-C.

(C) Rich club of the short-range aggregate network considering 100 nM EC_{50} threshold. The rich club coefficient $\Phi(k)$ for the *C. elegans* neuropeptidergic network is shown in black and the randomized rich club curve $\Phi_{random}(k)$ is depicted in gray. The red curve is the normalized coefficient $\Phi(k) \geq \Phi_{random}(k) + 10\sigma$ over the range $200 \leq k$, indicating the rich club. This means the short-range aggregate neuropeptide network considering 100 nM EC_{50} threshold has a rich club of 130 neurons, showing conservation of the rich club found with 500 nM EC_{50} threshold (Table S6). Related to Figure 6F and Table S6.

(D) Mid-range aggregate network at 100 nM EC_{50} threshold sorted in both dimensions (sending and receiving neurons) based on neuronal clusters defined in Figure 7A and Figure 7B. Neuronal cluster divisions are highlighted with continuous lines. Related to Figure 7F for comparison.

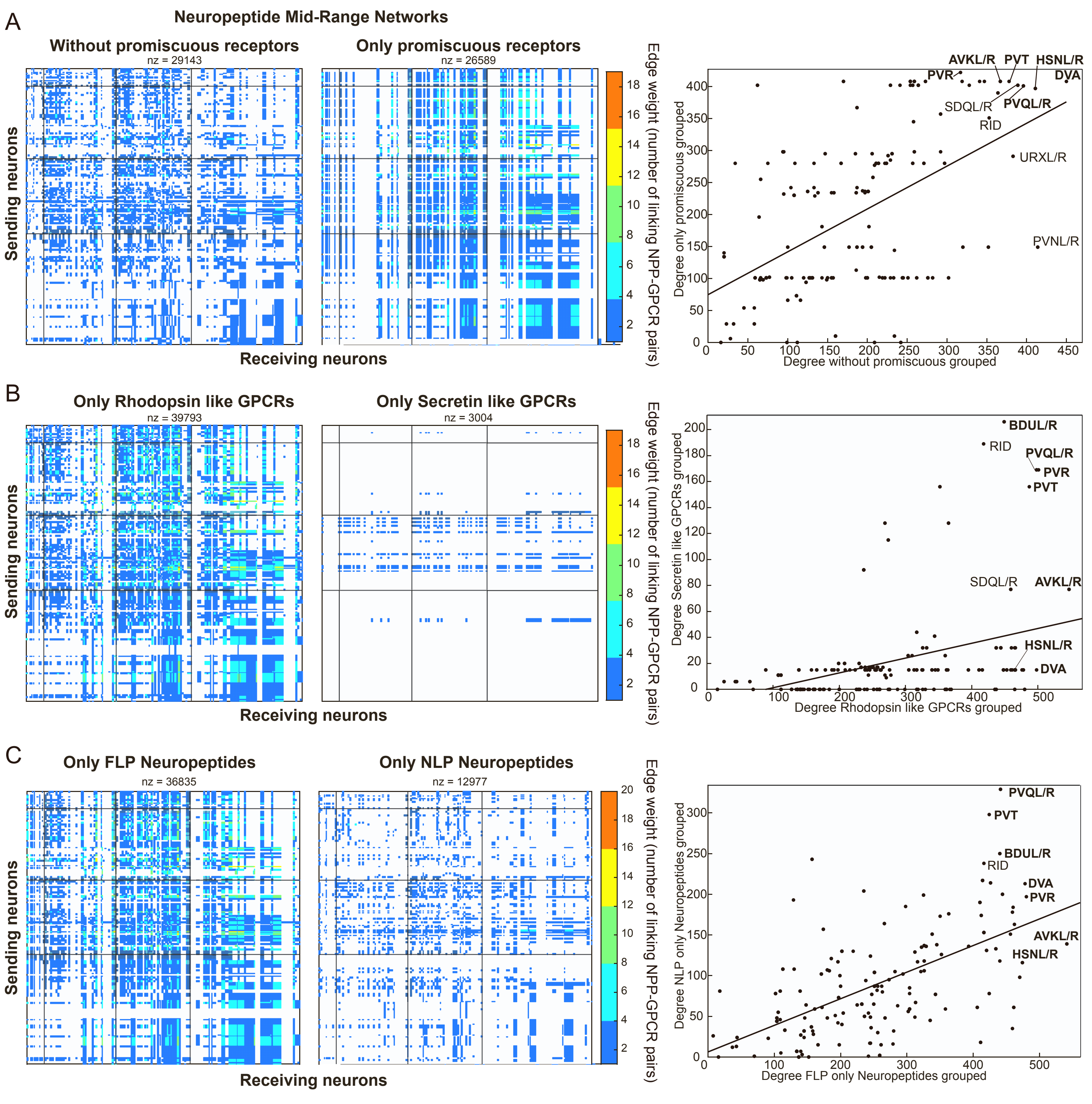
A
Neuropeptide Mid-Range Networks
Without promiscuous receptors
nz = 29143
Only promiscuous receptors
nz = 26589
Sending neurons
Receiving neurons
Edge weight (number of linking NPP-GPCR pairs)
AVKL/R
PVT
HSNL/R
PVR
DVA
SDQL/R
PVQL/R
RID
URXL/R
PVNL/R
Degree only promiscuous grouped
Degree without promiscuous grouped
B
Only Rhodopsin like GPCRs
nz = 39793
Only Secretin like GPCRs
nz = 3004
Sending neurons
Receiving neurons
Edge weight (number of linking NPP-GPCR pairs)
BDUL/R
RID
PVQL/R
PVR
PVT
SDQL/R
AVKL/R
HSNL/R
DVA
Degree Secretin like GPCRs grouped
Degree Rhodopsin like GPCRs grouped
C
Only FLP Neuropeptides
nz = 36835
Only NLP Neuropeptides
nz = 12977
Sending neurons
Receiving neurons
Edge weight (number of linking NPP-GPCR pairs)
PVQL/R
PVT
BDUL/R
RID
DVA
PVR
AVKL/R
HSNL/R
Degree NLP only Neuropeptides grouped
Degree FLP only Neuropeptides grouped

Figure S8. Partitioning the mid-range aggregate neuropeptide network based on neuropeptide system and receptor class. Related to Figure 5 and 6C. Adjacency matrix representations and correlation of degree distributions of partitioned neuropeptide networks considering the mid-range spatial diffusion models. For each degree distribution, the hubs with the highest neuropeptide degree in the overall mid-range neuropeptide network are highlighted in bold (AVKL/R, PVT, PVQL/R, PVR, DVA, BDUL/R, and HSNL/R), as defined in Figure 6C, are labeled for comparison. Other prominent neurons are also pictured. The number of connections (nz) is shown at the top of each matrix.

(A) Aggregate mid-range diffusion NPP-GPCR pair network considering all selected NPP-GPCR pairs (i.e. without promiscuous receptors - left), or only considering promiscuous NPP-GPCR pairs (middle), and the correlation of the total neuropeptide degree for each neuron between both networks (right).
(B) Aggregate mid-range diffusion NPP-GPCR pair network considering all rhodopsin-like GPCRs (left), or secretin-like receptors (middle), and the correlation of the total neuropeptide degree for each neuron between both networks (right).
(C) Aggregate mid-range diffusion NPP-GPCR pair network considering all FLP-type NPP-GPCR pairs (left), or NLP-type NPP-GPCR (middle), and the correlation of the total neuropeptide degree for each neuron between both networks (right).

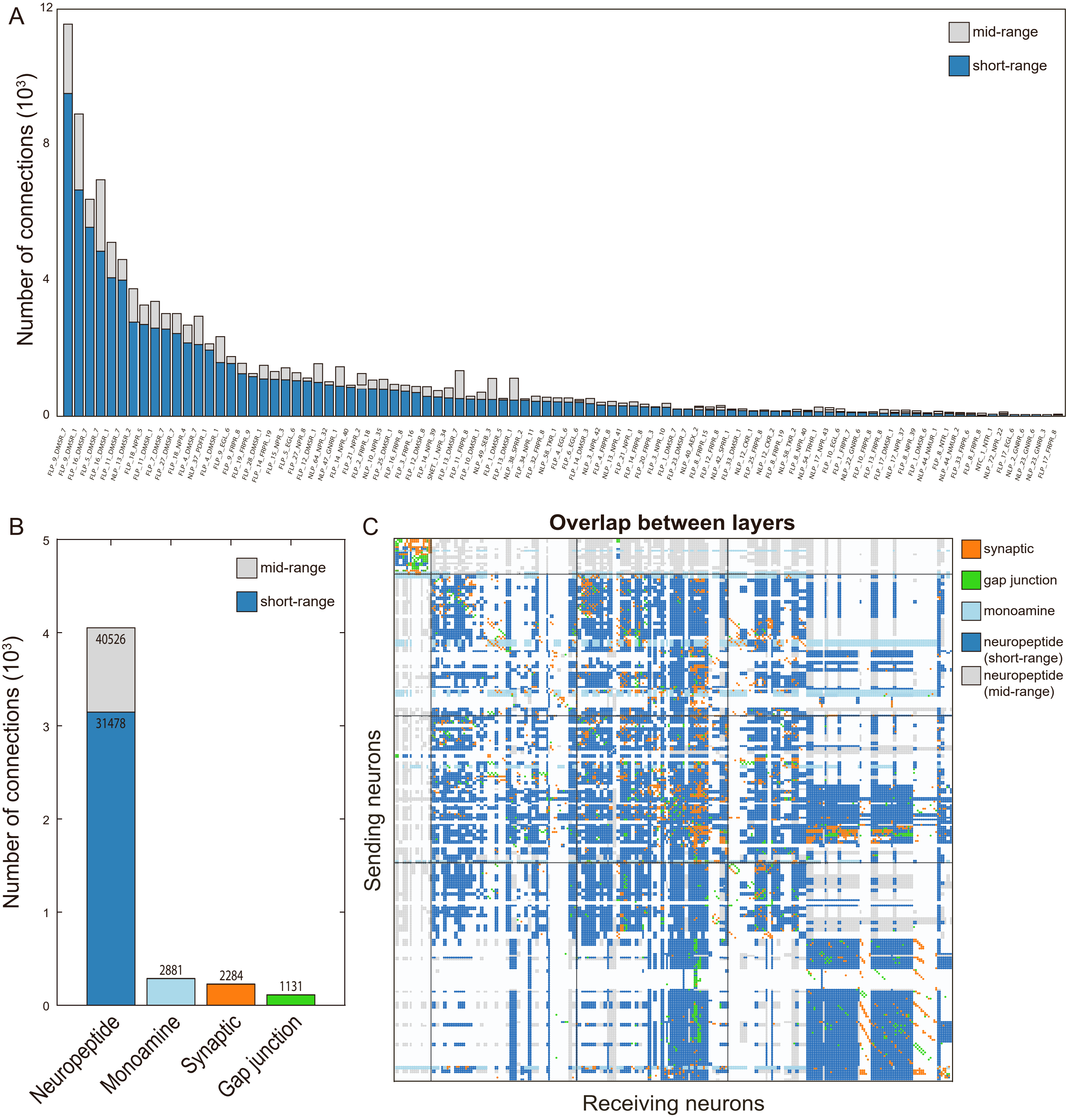

D

	NP mid-range	NP short-range	Monoamine	Synaptic	Gap Junction
NP mid-range	**40526**	31478	1446	1522	801
NP short-range		**31478**	1007	1521	798
Monoamine			**2881**	165	28
Synaptic				**2284**	253
Gap Junction					**1131**

Figure S9. Overlap between neuropeptide, monoamine, synaptic and gap junction networks within the *C. elegans* nervous system. Related to Figure 5.

(A) Bar plot showing the number of connections for each of the 92 individual NPP-GPCR pairs considering short-range (blue) and mid-range (gray) diffusion connections.
(B) Bar plot showing the number of connections for either neuropeptide (with short- or mid-range diffusion models, depicted in blue or gray, respectively), monoamine, synaptic, or synaptic and gap junction signaling.
(C) Adjacency matrix representation of the short-range neuropeptide (dark blue), mid-range neuropeptide (gray), monoamine (light blue), synaptic (orange) and gap junction (green) networks.
(D) Table depicting the number of connections that are shared between neuropeptide, monoamine, synaptic and gap junction signaling modalities.

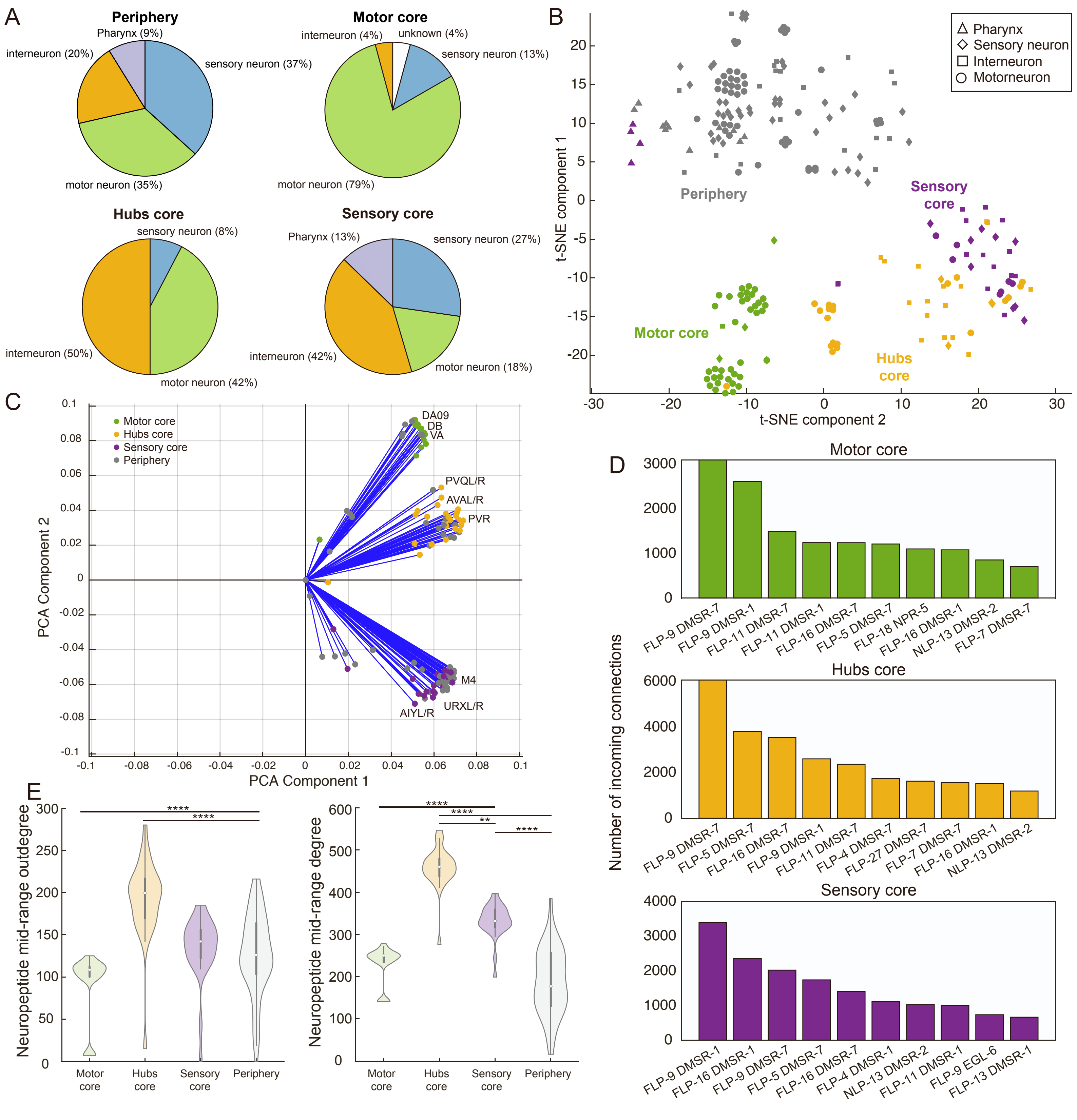

A
Periphery
Pharynx (9%)
interneuron (20%)
sensory neuron (37%)
motor neuron (35%)
Motor core
interneuron (4%)
unknown (4%)
sensory neuron (13%)
motor neuron (79%)
Hubs core
sensory neuron (8%)
interneuron (50%)
motor neuron (42%)
Sensory core
Pharynx (13%)
sensory neuron (27%)
interneuron (42%)
motor neuron (18%)
B
Pharynx
Sensory neuron
Interneuron
Motorneuron
Periphery
Sensory core
Motor core
Hubs core
t-SNE component 1
t-SNE component 2
C
Motor core
Hubs core
Sensory core
Periphery
DA09
DB
VA
PVQL/R
AVAL/R
PVR
M4
URXL/R
AIYL/R
PCA Component 2
PCA Component 1
D
Motor core
FLP-9 DMSR-7
FLP-9 DMSR-1
FLP-11 DMSR-7
FLP-11 DMSR-1
FLP-16 DMSR-7
FLP-5 DMSR-7
FLP-18 NPR-5
FLP-16 DMSR-1
NLP-13 DMSR-2
FLP-7 DMSR-7
Hubs core
FLP-9 DMSR-7
FLP-5 DMSR-7
FLP-16 DMSR-7
FLP-9 DMSR-1
FLP-11 DMSR-7
FLP-4 DMSR-7
FLP-27 DMSR-7
FLP-7 DMSR-7
FLP-16 DMSR-1
NLP-13 DMSR-2
Sensory core
FLP-9 DMSR-1
FLP-16 DMSR-1
FLP-9 DMSR-7
FLP-5 DMSR-7
FLP-16 DMSR-7
FLP-4 DMSR-1
NLP-13 DMSR-2
FLP-11 DMSR-1
FLP-9 EGL-6
FLP-13 DMSR-1
Number of incoming connections
E
Neuropeptide mid-range outdegree
Neuropeptide mid-range degree
Motor core
Hubs core
Sensory core
Periphery
****
**

Figure S10. Mesoscale structure of the neuropeptide connectome. Related to Figure 7.

(A) Pie charts showing the composition of the neuronal clusters defined in Figure 7 (A-B).
(B) t-SNE dimensionality reduction of the adjacency matrix of the short-range aggregate neuropeptide network (Euclidian distance, perplexity 30). Datapoint markers based on neuronal classification following t-SNE dimensionality reduction of the mid-range aggregate network of Figure 7A for comparison.
(C) Loading plot showing how individual neurons within the short-range aggregate network influence the two first principal components, related to Figure 7B. Datapoint markers based on the neuronal classification of Figure 7B. Neurons with the largest contribution are indicated.
(D) Number of incoming connections for the most prominent NPP-GPCR pairs in each cluster. Related to Figure 7B.
(E) Violin plots showing outdegree and total degree of the mid-range aggregate network for the four clusters, related to Figure 7D. Significances determined by Kruskal-Wallis test followed by Dunn-Sidak test for multiple comparisons with rank sums. n.s. not significant; $**p \leq 0.01$;$****p \leq 0.0001$.

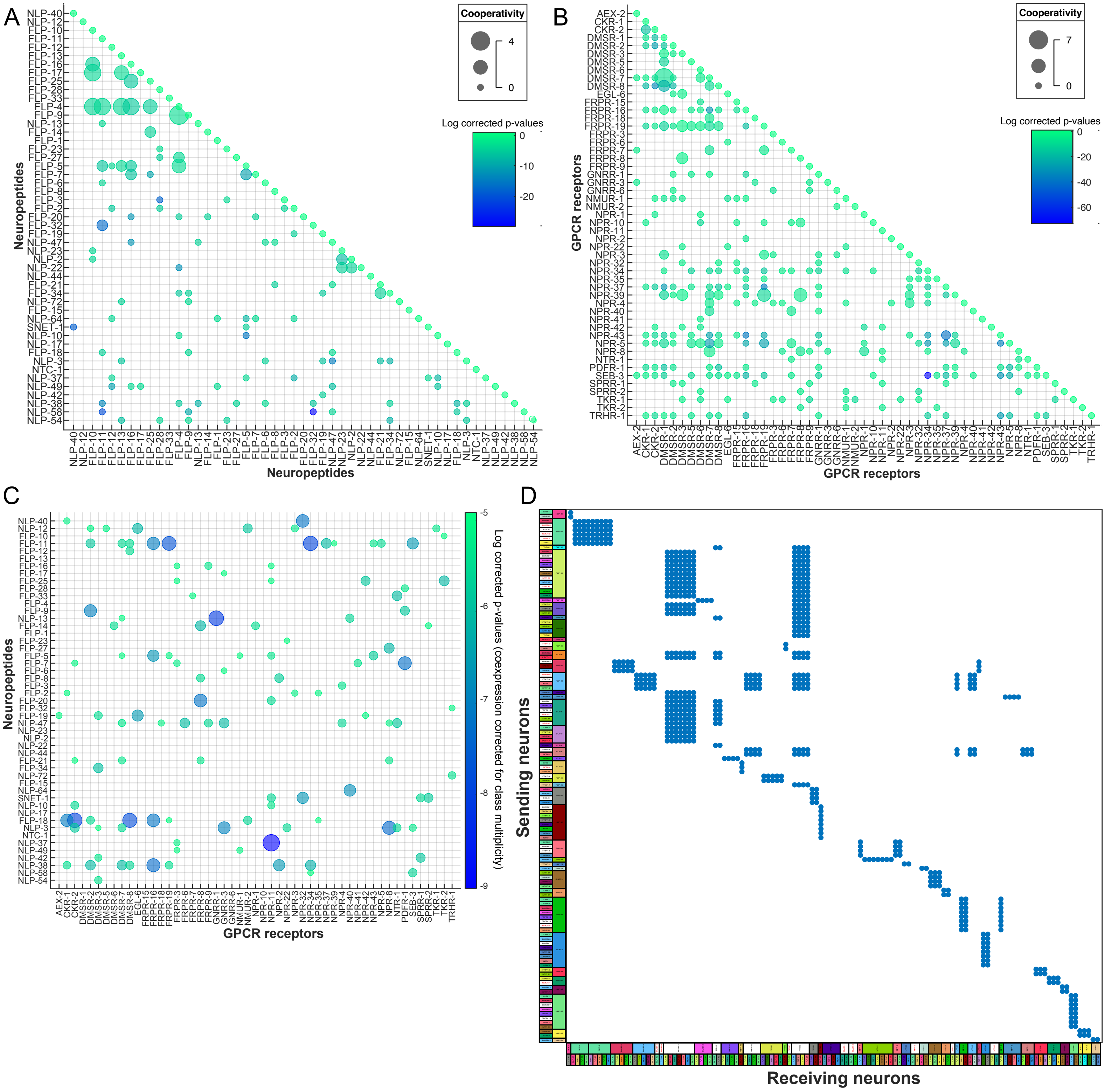
A
Cooperativity
4
0
Log corrected p-values
0
-10
-20
Neuropeptides
Neuropeptides
B
Cooperativity
7
0
Log corrected p-values
0
-20
-40
-60
GPCR receptors
GPCR receptors
C
Neuropeptides
GPCR receptors
Log corrected p-values (coexpression corrected for class multiplicity)
-5
-6
-7
-8
-9
D
Sending neurons
Receiving neurons

Figure S11. Co-expression analysis of NPP-GPCR non cognate pairs. Results of STAR Methods Co-expression analysis. Related to Figure 8.

(A) Bubble plot showing the Fisher's exact test association between the expression of two neuropeptide precursor genes (NPP) in the neuronal system of *C.elegans.* The color indicates the FDR (False Discovery Rate) corrected p-values for each NPP pair (log scale). The size of each bubble indicates the possible binding cooperativity between the two neuropeptides, from 0 (the two neuropeptides are not ligands to the same GPCR) to 4 (the two neuropeptides are ligands to the same 4 GPCRs). FLP-32/FLP-11 is the only example of high cooperativity and high expression association.

(B) Bubble plot showing the Fisher's exact test association between the expression of two GPCR genes in the neuronal system of *C.elegans.* The color indicates the FDR (False Discovery Rate) corrected p-values for each GPCR pair (log scale). The size of each bubble indicates the possible binding cooperativity between the two GPCRs, from 0 (the two GPCRs do not bind the same neuropeptides) to 4 (the two GPCRs bind the same 7 GPCRs).

(C) Bubble plot showing the Fisher's exact test association between the neuronal expression of the 51 GPCRs and 49 NPPs used in this study. The color indicates the FDR (False Discovery Rate) corrected p-values for each association (log scale). To prevent multiplicity effects due to the expression of the same genes in the same neuronal class, the values are corrected for neuronal class expression.

(D) Network of co-expression connections. In the y-axes the sending neurons are represented as the events of association between the neurons that express that neuropeptide and GPCRs from panel C. In the x-axes the receiving neurons are represented as the events of association between the neurons that express that GPCR and other neuropeptides from panel C. The different neuropeptide/GPCRs and GPCR/neuropeptides association events are labelled by colors, each GPCRs and their cognate neuropeptide are given the same color. The identities of these co-expression association events are shown in Figure 11S ID table.

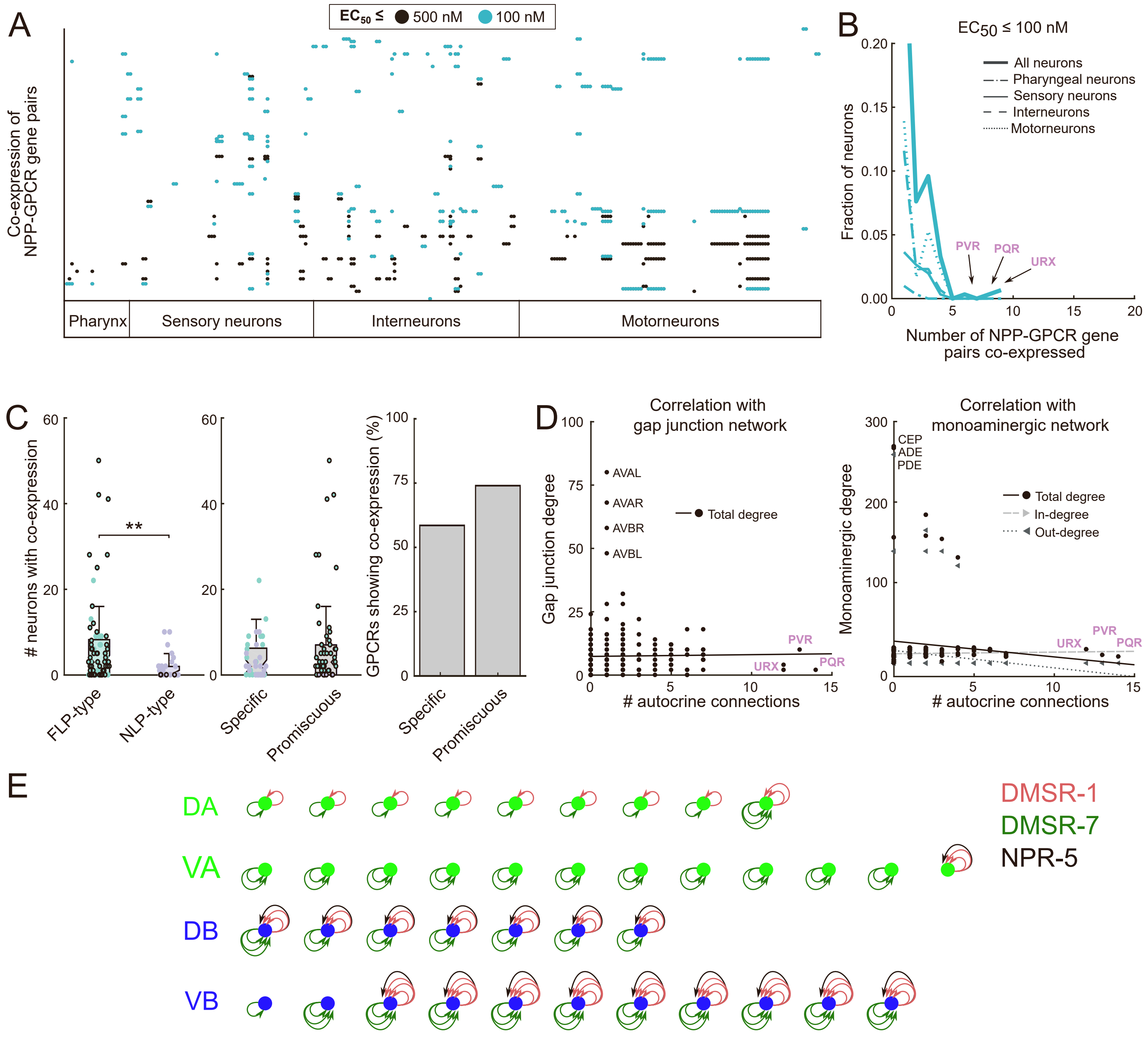
A
EC50 ≤ 500 nM 100 nM
Co-expression of NPP-GPCR gene pairs
Pharynx
Sensory neurons
Interneurons
Motorneurons
B
EC50 ≤ 100 nM
All neurons
Pharyngeal neurons
Sensory neurons
Interneurons
Motorneurons
Fraction of neurons
Number of NPP-GPCR gene pairs co-expressed
PVR
PQR
URX
C
neurons with co-expression
FLP-type
NLP-type
Specific
Promiscuous
GPCRs showing co-expression (%)
D
Correlation with gap junction network
Gap junction degree
AVAL
AVAR
AVBR
AVBL
Total degree
autocrine connections
Correlation with monoaminergic network
Monoaminergic degree
CEP
ADE
PDE
Total degree
In-degree
Out-degree
E
DA
VA
DB
VB
DMSR-1
DMSR-7
NPR-5

Figure S12. Autocrine connections. Related to Figure 8.

(A) Comparison of co-expression of cognate NPP-GPCRs pairs at 500 nM and 100 nM EC_{50} thresholds, depicted in gray and orange, respectively.

(B) Number of different NPP-GPCR pairs at 100 nM EC_{50} threshold being co-expressed in each neuron type. Neurons showing the highest diversity of different NPP-GPCR pairs being co-expressed are highlighted, including the sensory neurons PQR and URX and the interneuron PVR.

(C) Number of neurons with NPP-GPCR cognate pair co-expression for either FLP- or NLP-type neuropeptides show that FLP-type NPP-GPCR pair co-expression is more prevalent (left - Significances determined by Mann Whitney test; $^{**}p \leq 0.01$). Number of neurons with NPP-GPCR cognate pair co-expression for either specific or promiscuous GPCRs (middle). neuropeptides Percentage of NPP-GPCR co-expression events involving either specific or promiscuous GPCRs (right), suggesting that promiscuous GPCRs tend to have more autocrine connections.

(D) Correlation of number of autocrine connections with gap junction and monoamine networks.

(E) Number of autocrine connections in each A- and B-class motorneuron for each of the main promiscuous NPP-GPCR pairs present in those neurons.
